# An efficient transformation method for genome editing of elite bread wheat cultivars

**DOI:** 10.3389/fpls.2023.1135047

**Published:** 2023-05-16

**Authors:** Akshaya K. Biswal, L. Ruben B. Hernandez, Ana I. R. Castillo, Juan M. Debernardi, Kanwarpal S. Dhugga

**Affiliations:** ^1^International Maize and Wheat Improvement Center (CIMMYT), Texcoco, Mexico; ^2^Plant Transformation Facility, University of California, Davis, Davis, CA, United States

**Keywords:** wheat, transformation, GRF4-GIF1, particle bombardment, gene editing, CRISPR-Cas, Lr67, MLO

## Abstract

An efficient genetic transformation protocol is necessary to edit genes for trait improvement directly in elite bread wheat cultivars. We used a protein fusion between a wheat growth-regulating factor 4 (GRF4) and its interacting factor (GIF1) to develop a reproducible genetic transformation and regeneration protocol, which we then used to successfully transform elite bread wheat cultivars Baj, Kachu, Morocco, Reedling, RL6077, and Sujata in addition to the experimental cultivar Fielder. Immature embryos were transformed with the vector using particle bombardment method. Transformation frequency increased nearly 60-fold with the GRF4-GIF1-containing vectors as compared to the control vector and ranged from ~5% in the cultivar Kachu to 13% in the cultivar RL6077. We then edited two genes that confer resistance against leaf rust and powdery mildew directly in the aforementioned elite cultivars. A wheat promoter, TaU3 or TaU6, to drive the expression of guide RNA was effective in gene editing whereas the OsU3 promoter failed to generate any edits. Editing efficiency was nearly perfect with the wheat promoters. Our protocol has made it possible to edit genes directly in elite wheat cultivars and would be useful for gene editing in other wheat varieties, which have been recalcitrant to transformation thus far.

## Highlights

- We have developed an efficient protocol for the transformation and regeneration of fertile plants from elite bread wheat cultivars Baj, Kachu, Morocco, Reedling, RL6077 and Sujata as well as an experimental line Fielder.
- This protocol would be useful in editing genes directly in other wheat cultivars.

## Introduction

Wheat (*Triticum aestivum* L.) is a staple crop that provides ~20% of the global calorie needs and approximately 25% of daily protein intake (Hawkesford, 2014). A major portion of wheat yield is lost to biotic and abiotic stresses (Oerke, 2006; Wulff and Dhugga, 2018). Trait improvement through conventional breeding is a labor-intensive process that takes many years to complete. Gene editing directly in commercial cultivars is a viable alternative to expedite trait improvement, particularly for food security (Dhugga, 2022; Pixley et al., 2022). A recently introduced technology, Clustered Regularly Interspaced Short Palindromic Repeats and its associated endonucleases (CRISPR/Cas), has revolutionized the field of gene editing, which was slow to perform with the previously available technologies (Raghurami-Reddy et al., 2022). However, wheat lags other cereals such as rice and maize for its genetic manipulation through transformation, probably because of its complex genome. The very low regeneration efficiency even when it can be accomplished in elite wheat varieties is a bottleneck in gene editing (Bhalla, 2006; Garbus et al., 2015; Wulff and Dhugga, 2018).

Production of gene edited plants generally involves transformation, *in vitro* culture, and regeneration of transformed plants. Though transgenic plants were first reported as early as 1992 (Vasil et al., 1992), genetic transformation has been a significant impediment in wheat improvement (Hayta et al., 2019). In the last three decades, several protocols have been published for genetic manipulation of wheat through 1) biolistic particle bombardment (Vasil et al., 1992; Tassy and Barret, 2017; Ismagul et al., 2018; Miroshnichenko et al., 2018; Tanaka et al., 2022), 2) *Agrobacterium*-mediated transformation (Cheng et al., 1997; Wu et al., 2003; Ding et al., 2009; He et al., 2010; Ishida et al., 2015b; Hayta et al., 2019), 3) floral dip (Zale et al., 2009), 4) *in planta* particle bombardment (Liu et al., 2021), 5) *in planta Agrobacterium*-mediated inoculation (Supartana et al., 2006; Singh and Kumar, 2022), and 6) microspore transformation (Rustgi et al., 2017). These protocols had limited success in other laboratories, however. A high-efficiency *Agrobacterium*-mediated proprietary transformation protocol (PureWheat^®^ technology) was reported by Japan Tobacco group for the bread wheat cultivar Fielder (Richardson et al., 2014; Ishida et al., 2015a). This protocol requires optimization of multiple factors within narrow optimal windows without which the efficiency drops drastically (Ishida et al., 2015a). Despite repeated efforts, we could not replicate the protocol, as published, in our laboratory, an experience shared by other laboratories as well (Hayta et al., 2019; Debernardi et al., 2020).

Polyploidy often means editing more than one gene to improve a trait in wheat. For example, developing wheat powdery mildew resistant lines required simultaneous knocking out of all three homeologs of the *TaMLO* gene, which is a dominant suppressor of resistance (Wang et al., 2014). Editing of three homoeoalleles of protein disulfide isomerase like 5-1 (*TaPDIL5-1*) conferred resistance to wheat yellow mosaic virus (Kan et al., 2022). CRISPR/Cas9-mediated editing of genes in experimental wheat lines has already been reported. This, however, has limited application since transferring the edited gene to elite lines through breeding negates the advantage that gene editing offers.

Most of the current wheat transformation protocols are optimized for experimental lines like Fielder, Bob White, and Cadenza. Development of a fast and reliable genotype-independent transformation protocol is thus required to improve commercial cultivars directly. Recently, expression of a fusion protein of a wheat growth-regulating factor 4 (GRF4) and its cofactor, GRF interacting factor 1 (GIF1), significantly increased the regeneration efficiency of elite wheat lines (Debernardi et al., 2020). Introduction of a synonymous mutation in the miR396 target site of TaGRF4 was reported to further improve the performance of the mTaGRF4-TaGIF1 complex in comparison to the base TaGRF4-TaGIF1 complex in gene editing using a transient expression system (Qiu et al., 2022).

We used a binary vector containing the GRF4-GIF1 chimera to develop an efficient protocol to transform immature embryos of seven bread wheat cultivars using the particle bombardment method (Debernardi et al., 2020).

We developed constructs to knock out two adult plant resistance (APR) genes for rust and powdery mildew resistance. A single guide RNA (sgRNA) construct was designed to simultaneously knock out all three homoeologs of *Lr67* (Herrera-Foessel et al., 2011; Herrera-Foessel et al., 2014; Moore et al., 2015). For the second construct, we designed another sgRNA to knock out all three homeologs of wheat mildew locus O (*MLO*) in elite wheat cultivars (Piffanelli et al., 2002; Wang et al., 2014; Li et al., 2022). In this communication, we report a reproducible transformation protocol for six elite bread wheat varieties and a common spring wheat cultivar Fielder and simultaneous editing of three homoeologs of two wheat genes.

## Material and methods

### Plant growth and collection of immature embryos

Seeds of the spring wheat (*Triticum aestivum* L.) cv Fielder and elite cultivars Baj, Kachu, Reedling, RL6077 and Sujata were sown twice weekly in CIMMYT soil mix (7 portions of steamed recycled soil, 4 portions of steamed peat moss (Lambert^®^ Canadian Sphagnum, Canada), 1 portion of low-density sand) in 16-cm-diameter pots to maintain an uninterrupted supply of immature embryos. Plants were grown in our greenhouse at CIMMYT headquarters in Mexico (19° 31’ 47’’ N, 98° 50’ 44’’ W) at 23 ± 1°C Day and 18 ± 1°C night temperatures, 60-70% humidity and light levels of 500 μmol m^−2^ s^−1^ provided by direct sunlight and tungsten bulbs placed about 1 ½ meter above the plant tip. A 14/10-hour day/night cycle was maintained throughout the growth cycle by providing extra light in the evenings. Transgenic plantlets were initially grown in 10-cm-diameter pots in a controlled growth chamber (Conviron^®^ Europe Ltd, UK) in a 14/10-hour day/night cycle at 22 ± 1°C Day/night temperatures, 70% humidity, 500 μmol m^−2^ s^−1^ light during the day cycle. The plants were acclimatized by covering them with a transparent dome for at least 24 hours after transplantation. Seven to ten days after transplanting, well-established plantlets were transferred to 16-cm-diameter pots containing CIMMYT soil mix and were grown in the greenhouse in conditions mentioned above.

### Design of guide RNA

**Figure 1:**
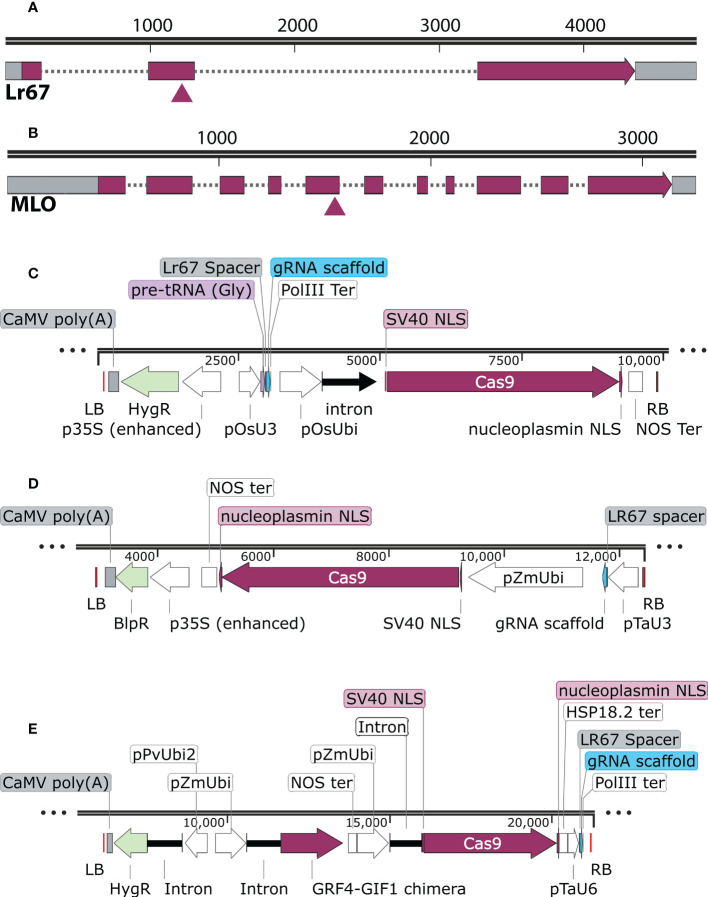
The relative location of the guide RNA in Lr67 and MLO genes and binary vectors used: **(A, B)** The triangle indicates the approximate location of guide RNA spacer in the second exon of *Lr67-4D* and the 5^th^ exon of *TaMLO-4B*. The spacers match the consensus sequences in all three homoeologs for both genes. **(C)** pRGEB32-derived vector in which the gRNA expression is driven by OsU3 promoter and rice Ubi promoter drives the *Cas9* expression, **(D)** pBun421-derived vector in which the gRNA expression is driven by TaU3 promoter and maize Ubi promoter drives the *Cas9* expression and **(E)** JD633-derived construct that carries the wheat GRF4-GIF1 chimera. Maize Ubi promoter drives the Cas9 expression and the gRNA expression is driven by TaU6 promoter. The vector maps were generated using Snapgene™ software V. 6.2 (www.snapgene.com).

We aimed to knock out all three homoeologs of *TaLr67* by targeting consensus regions in exon 2 as shown in **Figure 1A**. The gRNA spacers were designed using wheatCRISPR webtool (Cram et al., 2019). We also designed the gRNA targeting a unique site in the 5^th^ exon of *TaMLO* gene that was consensus to all three homoeologs (**Figure 1B**).

### Development of binary constructs

Because elements of the T-DNA often affect the transformation efficiency and expression of the transgene in the host, we tested three different binary vectors.

The pRGEB32 (Addgene plasmid #63142) vector system was designed to produce multiple gRNAs from a single polycistronic gene with tandemly arrayed tRNA–gRNA modules by using an endogenous tRNA-processing system, which precisely cleaves both ends of the tRNA precursor (Xie et al., 2015). This system can also be used to target a single gene. The *Cas9* gene with two nuclear localization signals is driven by a rice ubiquitin promoter plus the complete 5′-untranslated region (pOsUbi). The guide RNA (gRNA) is driven by rice U3 snoRNA promoter (pOsU3). Transformed calli and transgenic plants can be selected against hygromycin (Hyg). This vector system resulted in nearly 90% transformation efficiency for single gene editing in rice. Earlier, we successfully generated triple edited *Osxlg* mutants using this vector system (Biswal et al., 2022). The duplex guide oligo was inserted into the *BsaI* site of pRGEB32 using InFusion^®^ cloning at GenScript Biotech Inc., USA (**Figure 1C**). The final construct was verified by Sanger sequencing.

The pBun421 (Addgene plasmid # 62204) binary vector harbors a maize-codon optimized *Cas9* gene, which is also driven by maize Ubi promoter (pZmUbi) (Xing et al., 2014). The gRNA expression is controlled by wheat U3 promoter (pTaU3), which was reported to perform slightly better in gene editing efficiency than rice U3 promoter (Xing et al., 2014). Transformed calli and transgenic plants can be selected against phosphinothricin (Ppt). A pair of oligos, specific to the target sequence (19 nucleotides), were designed in such a manner that they generate a four-base pair (5’- agcg) overhang at the 5’-end and another four-base pair (caaa - 3’) overhang at the 3’-end (**Supplementary Table S1**). Both oligos were phosphorylated using T4 polynucleotide kinase and annealed to form the spacer dimer, which was cloned into the pair of *BsaI* sites of the pBun421 binary vector (**Figure 1D**). The final construct was verified by Sanger sequencing.

The JD633-GRF4-GIF1 vector was developed as mentioned by Debernardi et al. (2020). The T-DNA carries a *GRF4–GIF1* chimera under the control of the maize Ubiquitin promoter (pZmUbi) and is preceded by a *nos* terminator. The GRF4 and GIF1 ORFs are separated by a 12-nucleotide bridge of four alanine residues and have been shown to have optimal effect on regeneration. The wheat codon optimized Cas9 with a nuclear localization signal from nucleoplasmin is driven by a maize Ubi promoter (pZmUbi). The guide RNA (gRNA) is driven by wheat U6 promoter (pTaU6). Transformed calli and transgenic plants can be selected against hygromycin (Hyg). A pair of oligos, specific to the target sequence, were designed in such a manner that they generate a four-base pair (5’ - actt) overhang at the 5’-end and another four-base pair (caaa – 3’) overhang at the 3’-end. Both oligos were phosphorylated using T4 polynucleotide kinase and annealed to form the spacer dimer, which was later cloned into the pair of *AarI* sites of the binary vector to form the JD633-GRF4-GIF1 CRISPR vector (**Figure 1E**). We validated the JD633-GRF4–GIF1 CRISPR vector for each target gene by Sanger sequencing.

### Genetic transformation by particle bombardment

We initially used three different media and protocol combinations to regenerate plantlets after biolistic transformation of immature embryos (IEs) with a pRGEB32-based binary vector for *Lr67* gene editing construct. Ishida et al. (2015a) published an *Agrobacterium*-mediated transformation (PureWheat^®^) protocol showing 50 - 60% transformation efficiency in Fielder. We first attempted to regenerate plantlets from wheat IEs after particle bombardment using the PureWheat^®^ protocol. Another comprehensive *Agrobacterium*-mediated transformation protocol, earlier developed by the same team (Hiei and Komari, 2006; Hiei and Komari, 2008), was later optimized by Slamet-Loedin et al. (2014) for regeneration of rice plantlets from a wide range of rice genotypes. Since the key factors for improvement of wheat transformation and regeneration were similar to those for rice and maize, we, therefore, adopted the modified protocol of Slamet-Loedin et al. (2014), to regenerate plantlets post-particle bombardment. Jordan (2000) used a jellyfish (*Aequorea Victoria)* green fluorescent protein (GFP) to identify stable transformants beginning as early as 21 days after transformation of wheat IEs by particle bombardment. We implemented this protocol to develop our transformation protocol with certain modifications as described below.

#### Preparation of gold particles

Ten mg of 0.6 µm gold particle (Bio-Rad, USA Cat. # 1652262) was measured into a 1.5 ml microfuge tube and 1 ml of 95% (V/V) ethanol was added to it. Mixed well by vortexing and centrifuged for 30 sec at 1000 x g (~3000 rpm) in a tabletop centrifuge. The ethanol supernatant was carefully pipetted out while keeping the tear-drop shaped pellet facing down to avoid pipetting of gold particles. The particles were washed twice with 1 ml aliquots of sterile ddH_2_O. Care was taken to mix the particles well each time by vortexing. The gold particles were resuspended well by vortexing in 500 µl of 50% (V/V) sterile glycerol prepared with sterile ddH_2_O and quickly aliquoted into 1.5ml sterile microfuge tubes (25 µl each). To keep the mixture uniform, vortexing was repeated after every 3 withdrawals. The gold particles were stored in a –20°C freezer till further use.

#### Harvesting and sterilization of immature seeds

Spikes were harvested 13 days after anthesis (DAA). Depending on the cultivar and size of immature embryo, this can be relaxed up to 15 DAA. Immature wheat kernels were extracted from the spikelet, put in 50 ml falcon tubes, and rinsed with distilled water for a minute. Intact kernels were then washed with 70% ethanol for a minute followed by surface sterilization using 25% bleach (containing ~1.5% Sodium hypochlorite) with 2 drops of Tween 20 and by rotating in a rotor for 10 minutes. The kernels were rinsed five times with sterile distilled water for one minute each to remove excess bleach.

#### Extraction of immature embryos

Immature embryos (IE) (1.5-2.0 mm) were extracted aseptically under a light microscope in a laminar airflow cabinet. The radical axis was removed with the help of a No. 11 scalpel, and IEs were placed on callus induction media (**Table 1**) with 2-3 mm gap between embryos. After completion of IE extraction, 25 IEs were arranged at the center of a 60 mm petri dish containing osmotic media (**Table 1**), with their scutellum side facing up without leaving any space between them and were left in the laminar airflow cabinet for 3 hours. A separate plate was prepared as a control for each experiment.

**Table 1:** Media composition.

Reagent	Osmoticum (1 L)	Callus Induction (1 L)	Selection (1 L)	Regeneration (1 L)	MS (1L)	½ MS (1L)
MS Macrosalts [10x] ^*^	200 ml	200 ml	200 ml	–	–	–
MS Microsalts [1000x] ^*^	2 ml	2 ml	2 ml	2 ml	–	–
L7 Macrosalts [10X] ^*^	–	–	–	200 ml	–	–
10X MS^*^	–	–	–	–	100 ml	50 ml
MS FeNaEDTA [100X] ^*^	20 ml	20 ml	20 ml	20 ml	–	–
MS Vitamins/Inositol[1000x] ^*^	2 ml	2 ml	2 ml	2 ml	–	–
L-Glutamine	0.75 g	0.75 g	0.75 g	–	–	–
L-Proline	0.15 g	0.15 g	0.15 g	–	–	–
L-Asparagine	0.1 g	0.1 g	0.1 g	–	–	–
Sucrose	–	60 g	60 g	–	30 g	15 g
Maltose	200 g	–	–	60 g	–	–
MES	3.9 g	3.9 g	3.9 g	–	–	–
Glucose	10 g	–	20 g	–	–	–
MgCI2·7H2O	1.5 g	–	–	–	–	–
pH	5.2	5.7	5.7	5.7	5.8	5.8
After autoclave, add						
2,4-D [1mg/ml water]		1.0 ml	1.0 ml	–	–	–
Picloram [1mg/ml water]		1.0 ml	1.0 ml	–	–	–
Zeatin [5mg/ml water]	–	–	–	1.0 ml	–	–
Phytagel [5.0g/L] (pre-autoclaved))		500 ml	500 ml	500 ml	4g	4g
Hygromycin [50mg/ml]	–	–	0.6 ml	0.6 ml	0.6 ml	0.6 ml

^*^: Composition of these stocks has been given in **Table 2**.

#### Preparation of macrocarriers with DNA/gold particle (5-6 bombardments)

High concentration (500 ng/µl – 1 µg/µl) plasmid DNA was extracted by mini-prep using QIAprep Spin Miniprep Kit^®^ (Qiagen, USA) as per the supplier’s user manual. One vial of gold particle was taken out from the freezer and thawed by leaving for 1-2 minutes in the laminar airflow cabinet. Three to five µg (max. 25% V/V) of plasmid DNA was added to each vial of gold particles and mixed well by finger-tapping. (Note: If using 2 plasmids, add 2.5 -3.5µl of each one). Ten µl of 0.1 M spermidine (dissolved in ddH_2_O, filter-sterilized and stored at –20°C until use) was added to the side of the microfuge tube so that it did not mix with the DNA. Then 25 µl of 2.5 M CaCl_2_ (dissolved in ddH_2_O, filter-sterilized and stored at –20°C until use) was added to a different location on the wall of the microfuge tube. The vial was flicked, finger tapped immediately and incubated for 20 minutes at RT in the laminar airflow cabinet for binding of DNA to the gold particles. While incubating the DNA/Gold particles, macrocarriers were sterilized by dipping in 95% ethanol for 2-3 minutes and installed into sterile (autoclaved) microcarrier holders using a blunt forceps.

Subsequently, the microfuge tubes were centrifuged for 30 sec at 1000 x g (~3000 rpm) in a tabletop centrifuge. The supernatant was removed, and the DNA-coated particles were resuspended in 200 µl of 70% (V/V) ethanol by tapping with finger. The tubes were spinned as above, the supernatant was removed, and the DNA-coated particles were resuspended in 200 µl of 95% (V/V) ethanol by finger tapping. The spinning was repeated, supernatant was removed, and the DNA-coated particles were finally resuspended in 50 µl of 95% (V/V) ethanol as above. Quickly, 5 µl of DNA-coated particle solution was loaded onto the center of the macrocarrier already installed in the microcarrier holder and allowed to air-dry completely in the laminar air-flow cabinet.

#### Particle bombardment

The regulator of the helium gas was adjusted to achieve 200 psi over desired rupture pressure (ie. 1100 psi for 900 psi rupture disks^®^ that we typically use (Cat. no. 1652328, Bio-Rad, USA). The sample chamber was wiped with 70% ethanol. The vacuum pump and gene gun (Bio-Rad, USA) were turned on sequentially. The rupture disk was sterilized by soaking in 2-Propanol for 3-5 min and then placed inside the rupture disk holder that was subsequently screwed onto the end of the gas tube. The stopping screen (sterilized by autoclave) was placed inside microcarrier launch assembly using sterile forceps and the macrocarrier holder was placed on it keeping the DNA-face down. The launch assembly was closed by screwing the cover lid until snug and was inserted into the slot directly below the rupture disk holder (into the second slot from the top of the gun chamber). A 60 mm petridish carrying the IEs at the center (without lid) was loaded to the third rack from the bottom of the gene gun chamber and the IEs were aligned with the hole of the launch assembly. The door was latched gently, and the vacuum was turned on. When vacuum reached the desired level (the fire button light turns on), the fire button was pressed and held until the rupture disk was burst (it bursts within 100 psi of desired pressure). The fire button was released quickly and then the vacuum switch was turned to the vent position (the fire button light goes off). The IE plate was removed and kept in the laminar airflow cabinet. The rupture disk particles and macrocarrier were discarded while the stopping screen could be reused up to three shots (while using the same plasmid construct). The bombardment was repeated once again for each plate as above, and then the plate was incubated at 25°C in the incubator (Conviron, UK) overnight in dark.

### Tissue culture and regeneration of plantlets

#### Callus induction

**Figure 2:**
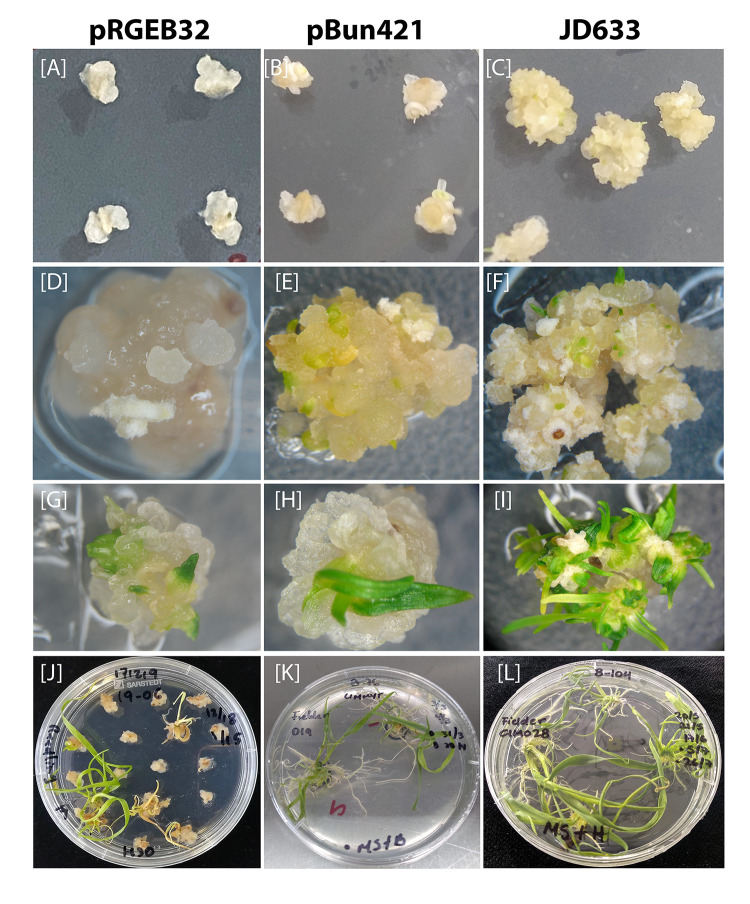
Comparison of different post-transformation tissue culture stages using constructs derived from thee different binary vectors: **(A–C)** Calli generated from 13-DAA immature embryos (IEs) after two cycles of callus induction (28 days without selections plus 14 days with the selection agent) with various vectors, **(D–F)** development of embryogenic calli after two weeks of regeneration, **(G–I)** shoots regenerated from the calli and **(J–L)** generation of plantlets. Note: The pRGEB32 plates carry a greater number of calli per plate since initial standardization started with 16-18 IE/calli per plate.

The next day (after ~24 hours of particle bombardment), the IEs were transferred (scutellum side up) to callus induction media (**Table 1**) without antibiotics in 90 mm petri-dish (25 embryos/plate) and incubated in the dark at 25°C for 28 days in the growth chamber (Conviron, Inc., UK) by when embryogenic clumps or pro-embryogenic callus could be observed (**Figures 2A–C**). Then the calli were transferred to fresh petri dishes (14 calli/plate) containing selection media (**Table 1**) with appropriate antibiotics (30 mg/L Hyg, or 5 mg/L Ppt) and were incubated in the dark at 25°C for 14 days in the growth chamber for selection of transformed calli. Many of the transformed calli become bigger and turn into embryogenic (whitish) calli (**Figures 2D–F**).

#### Regeneration (in tissue culture room at 24°C)

The embryogenic calli were acclimatized in the light for 3 days at 24°C in a low light area of the tissue culture room before transferring them to the regeneration media (we usually put them on a lower rack without switching on the light for that rack). Green somatic embryos could be easily observed after 3 days. Calli with green somatic embryos were transferred to 90 mm petridishes (9 calli/plate) with regeneration medium (**Table 1**) containing appropriate antibiotics (30 mg/L Hyg, or 5 mg/L Ppt) and were incubated under white florescent light (100 μmol. m^−2^. s^−1^) for 21 days with 16 h photoperiod.

#### Rooting (in tissue culture room at 24°C) and transplantation

Calli with small green shoots (**Figures 2G–I**) were transferred to 90 mm petri dish with MS medium (**Table 1**) plus appropriate antibiotics (30 mg/L Hyg, or 5 mg/L Ppt) (4 calli/plantlets per plate) and incubated under white florescent light (100 μmol. m^−2^. s^−1^) for 14 days with 16 h photoperiod (we try to remove most of the calli from the plantlet without affecting the plantlet and root, if any). Afterwards, plantlets with green leaves and profuse long roots were transferred into 50 ml falcon tubes with ½ MS medium (**Table 1**) supplemented with 30 mg/L Hyg or 5 mg/L Ppt for 10-14 days (while transferring, a small piece of leaf was taken for PCR analysis); only one plantlet per calli was transferred into rooting media to maintain independency of transgenic events. Well-rooted plants were gently removed from the tubes using long forceps, roots were gently washed with cool running tap-water to remove any remaining tissue culture medium and transferred into soil in 4-inch square pots and placed in the growth chamber at 22°C with 16 h photoperiod. The plants were covered with a transparent dome for at least 24 hours.

### Molecular characterization

#### Screening for transgene integration by PCR

The genomic DNA was extracted from 2 to 3-cm-long fresh and young leaves by using a modified CTAB protocol (Rajendrakumar et al., 2007). The plasmid integration was analyzed by PCR on genomic DNA using two sets of primers for each construct (**Supplementary Table S2**). Reactions were performed in 20-µl volume containing 1x PCR buffer (10 mM Tris-HCl [pH 8.3], 50 mM KCl, 1.5 mM MgCl_2_, 0.01% [v/v] gelatin), 25 to 50 ng of template DNA, 2 pmol of each primer, 250 µmol of each deoxyribonucleotides (dNTPs), and 1 unit of GoTaq^®^ DNA polymerase (Promega, USA). The initial denaturation was performed at 95°C for 3 min followed by 30 cycles of a thermal profile comprising denaturation at 95°C for 20s, annealing for 20s at primer-specific temperature (**Supplementary Table S2**) and extension for 1 min at 72°C. The final extension was performed for 5 min at 72°C. All PCR products were separated on 1% (w/v) agarose gels (Seakem LE agarose, Lonza Bioscience, USA), stained with SYBR Safe gel stain (Thermofisher, USA) and documented in a gel doc (Bio-Rad, USA).

#### Determination of transgene copy number by real-time quantitative PCR

The transgene copy number was determined by real-time quantitative PCR (qPCR) as described by Shepherd et al. (2009). The qPCR reactions were performed in an ABI 7500 Fast real-time PCR system (ThermoFisher Scientific, USA) using primer pairs TaU6_851F/pRGEB_gRNAR for the transgene and Lr67_1369F/Lr67OEP_2R for the *Lr67* gene (reference gene) that is known to be present in three copies in the wheat genome (**Supplementary Table S2**). Each reactions (20 μl) contained 10.0 μl of 2x Luna universal qPCR master mix (New England Biolabs, USA), 5.0 μl of nuclease free water, 5.0 pmol each for the primers and 4 µl of genomic DNA. The cycling parameters were 95°C for 1 min for initial denaturation followed by 45 cycles of 95°C for 15 s and 60°C for 30 s. The melt curve analysis was performed at 1% ramp rate. Each reaction was run in triplicate.

#### Mutation analysis

Target sequences were amplified by Phusion DNA polymerase (New England Biolabls, United States) using gene-specific primers flanking the target site (**Supplementary Table S3**) using the protocol mentioned by Biswal et al. (2022). The amplicons were initially analyzed by Surveyor mutation detection assay (IDT, USA) as per supplier’s instruction to detect mutants and their zygosity. Mutations were further confirmed by Sanger sequencing. The chromatograms were analyzed by Inference of CRISPR Edits (ICE) analysis (Conant et al., 2022) or CRISP-ID (Dehairs et al., 2016).

### Statistical analysis

We transferred only one plantlet per calli into rooting media to maintain all subsequent plants as independent transformation events. The regeneration efficiency was calculated by the formula:


$$Regeneration\;efficiency=\frac{\text{No}.\text{ of calli that regenerated into plantlets}}{\text{Number of IEs bombarded}}\;\times 100\%$$

The transformation efficiency was calculated by the formula:


$$Transformation\;efficiency=\frac{\text{Number of PCR positive plants}}{\text{Number of IEs bombarded}}\;\times 100\%$$

All statistical analyses were performed using Prism 9.5.0 (GraphPad Software, LLC, USA). The significance of differences was analyzed using a two-way ANOVA followed by Tukey’s honestly significant difference (HSD).

## Results

### Callus induction and somatic embryogenesis

In comparison to rice or maize, wheat calli grow at a slower pace. An initial incubation of four weeks in the dark without the selection agent followed by a second incubation for two weeks in the dark in the presence of the selection agent resulted in large embryogenic calli (**Figures 2A, B**). However, transformation with plasmids carrying the GRF4-GIF1 chimera resulted in larger and more embryogenic calli than those without GRF4-GIF1 for all the cultivars (**Figure 2C**).

### Regeneration of plantlets

The biolistic particle bombardment method has been successfully used to generate stable transgenic wheat plants despite low regeneration efficiency. We performed an initial study to examine the effect of different phytohormones and optimize the conditions for regeneration. For each of three transformation protocols, which are mentioned in the material and methods section, we transformed 4,000-6,000 IEs of cultivars Fielder and Reedling. The *in vitro* culture recommendations of both the PureWheat^®^ technology (Ishida et al., 2015a) and the optimized rice protocol of Slamet-Loedin et al. (2014) failed to generate stable transgenic events from IEs after particle bombardment. However, we were able to generate a total of eight independent transgenic events with our modified protocol (**Figure 2J**). All subsequent transformations were performed using this protocol.

Constructs derived from pRGEB32 vector system generated only a few embryogenic calli among a large number of watery, non-embryogenic calli after two cycles of callus induction and selection (**Figure 2D**). Only a few of these calli regenerated into plantlets (**Figures 2G, J**), and none was edited for the target gene (discussed in the next section). Thus, we discontinued further evaluation of the regeneration and transformation efficiencies for this vector. Constructs derived from pBun421 vector produced relatively more embryogenic calli than pRGEB32 (**Figures 2E, H**), but the frequency of plantlet regeneration was still low (**Figure 2K**). The JD633-based vector containing the GRF4-GIF1 chimera significantly improved the regeneration efficiency as compared to the other vectors (**Figure 2L**).

**Figure 3:**
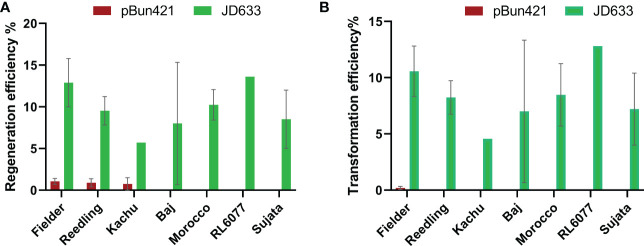
Significant improvement in **(A)** regeneration and **(B)** transformation efficiencies by the GRF4-GIF1 based vectors. A two-way ANOVA was performed to analyze the source of variation. The p-value for the row-factor (cultivar variation) was 0.9414 (ns) and 0.9443 (ns) whereas the column factor (variation in the vector backbone) was <0.0001 (significant) and <0.0001 (significant) for regeneration and transformation respectively. We did not transform Morocco, RL6077 and Sujata using the pBun421-based vectors. Hence, we did not include these lines for statistical analyses.

We then tested pBun421-based and JD633-based vectors in multiple cultivars. For the pBun421-based vectors, the respective regeneration efficiencies were 1.1 ± 0.35% for Fielder, 0.89 ± 0.49% for Reedling, and 0.75 ± 0.75% for Kachu. We were unable to regenerate plantlets for Baj using these vectors. The GRF4-GIF1 chimera in JD633-based vectors, in contrast, significantly improved the regeneration of all three cultivars (**Figure 3A**). The regeneration efficiency of Kachu increased to 5.7 ± 0.0%, Reedling to 9.52 ± 1.7% and that of Fielder to 12.9 ± 2.9% (**Figure 3A** and **Table 2**). For some batches, the regeneration efficiency was nearly 60%. Further, we were successful in regenerating plantlets for the cultivar Baj (8.0 ± 7.3%) using the JD633-based vectors. Later, we regenerated several plantlets for the cultivars Morocco, RL6077 and Sujata using the JD633-based vectors (**Table 3**, **S4** and **Figure 3A**).

**Table 2:** Composition of stock solutions.

Stock solution	Component	Quantity/L	Note
10X MS Macrosalts	NH_4_NO_3_	16.5 g	First dissolve CaCl_2_·2H_2_O in distilled water at 70% of the volume to be prepared before adding the other ingredients and then add the remaining ingredients. Make up the vol and autoclave. Store at 4°C
	KNO_3_	19 g	
	KH_2_PO_4_	1.7 g	
	MgSO_4_·7H_2_O	3.7 g	
	CaCl_2_·2H_2_O	4.4 g	
1000X MS Microsalts	MnSO_4_ *	10 g	*If MnSO_4_ is not available use MnSO_4_·H_2_O (11.37 g), MnSO_4_·4H_2_O (15.48 g) or, MnSO_4_·7H_2_O (18.63 g). Sterilize by autoclaving and store at 4°C
	H_3_Bo_3_	6.2 g	
	ZnSO_4_·2H_2_O	5.8 g	
	KI	0.8 g	
	Na_2_MoO_4_·2H_2_O	0.25 g	
	CuSO_4_·5H_2_O	0.025 g	
	CoCl·6H_2_O	0.025 g	
10X MS	MgSO_4_ *7H_2_O	3.70 g	Divide into 50 ml aliquots and store at -20°C ^#^Store at 4°C ^##^Store at –20°C
	MnSO_4_ *H_2_O	169 mg	
	CaCl_2_ *2 H_2_O	4.35 g	
	NH_4_NO_3_	16.5 g	
	KNO_3_	19.0 g	
	KH_2_PO_4_	1.7 g	
	H_3_BO_3_	62 mg	
	ZnSO_4_*7H_2_O	86 mg	
	Fe (III) EDTA	425 mg	
	Myo-Inositol	1.0 g	
	CuSO_4_*5H_2_O [0.25 mg/ml]^#^	1.0 ml	
	KI [7.5 mg/ml] ^#^	1.0 ml	
	CoCl_2_*6H_2_O [0.25 mg/ml)] ^#^	1.0 ml	
	Na_2_MoO_4_*2H_2_O [2.5 mg/ml] ^#^	1.0 ml	
	Nicotinic Acid [5.0 mg/ml]^##^	1.0 ml	
	Pyridoxine (B6) [5.0 mg/ml] ^##^	1.0 ml	
	Thiamine (B1) [1.0 mg/ml] ^##^	1.0 ml	
1000X MS Vitamins/Inositol (-Glycine)	Thiamine.HCl	100 mg	Sterilize by autoclaving and store at 4°C
	Pyridoxine.HCI	500 mg	
	Nicotinic acid	500 mg	
	Inositol	2.0 g	
MS - FeNaEDTA	FeSO_4_.7H_2_O	2.785g	Store at 4°C
	Na_2_EDTA.2H_2_O	3.725 g	
10X L7 Macrosalts	NH_4_NO_3_	2.5 g	Store at 4°C
	KNO_3_	15.0 g	
	KH_2_PO_4_	2.0 g	
	MgSO_4_·7H_2_O	3.5 g	
	CaCl_2_·2H_2_O	4.5 g	

**Table 3:** Average regeneration efficiency of different cultivars after transformation with pBun421 and JD633 (carrying GRF4-GIF1 growth regulation chimera).

Cultivar	PBun421 plasmids			GRF4-GIF1 carrying plasmids		
	Mean^*^	SEM	N^#^	Mean^*^	SEM	N^#^
Fielder	1.06	0.35	22	12.9	2.9	20
Reedling	0.89	0.49	3	9.52	1.70	9
Kachu	0.75	0.75	2	5.71	0.00	1
Baj	0.00	0.00	2	8.00	7.33	2
Morocco	NA			10.24	1.83	3
RL6077	NA			13.6	0.00	1
Sujata	NA			8.50	3.5	2

^*^Mean regeneration efficiency values are significantly different between PBun421 plasmids and GRF4-GIF1 carrying plasmids for the same cultivar (P<0.0001).

^#^N: Number of experimental batches with 100 – 200 calli per batch

NA: No experiment was performed.

### Transgene integration and transformation efficiency

**Figure 4:**
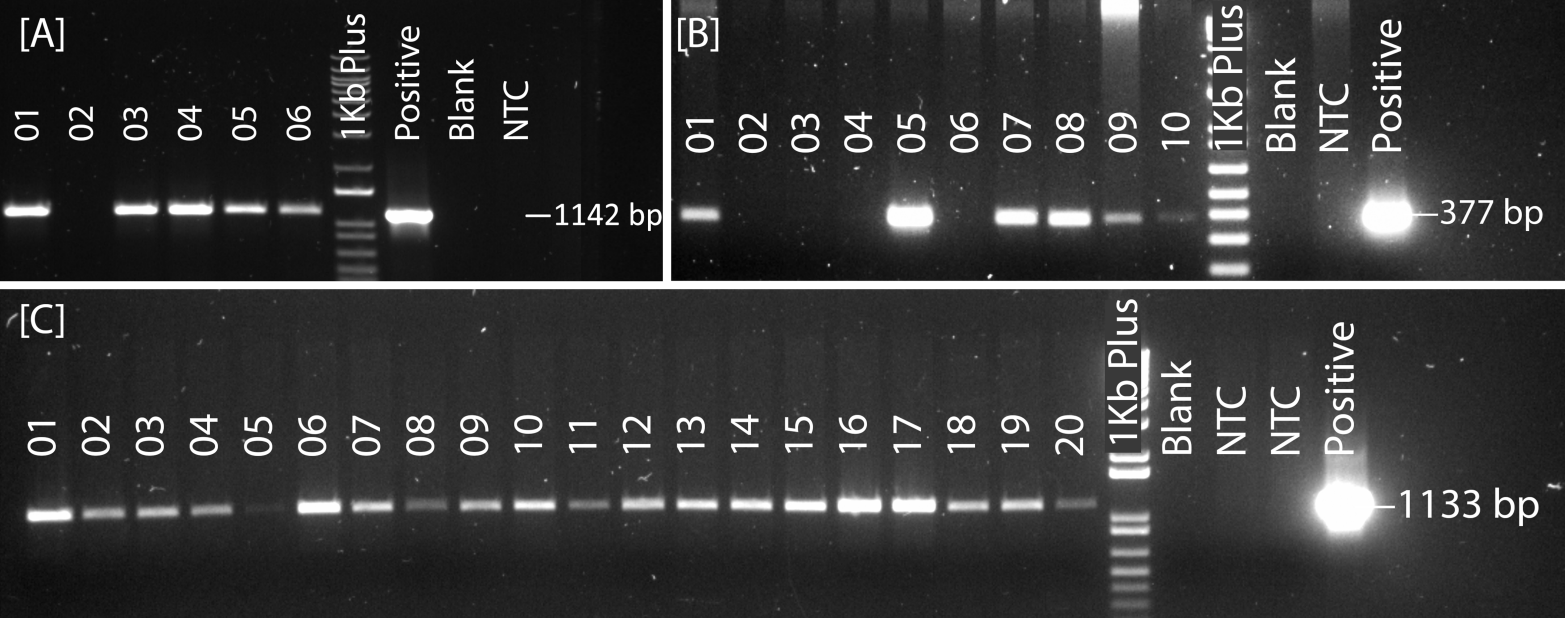
PCR screening of plasmid integration in plantlets obtained after transformation with the derivatives of the following vectors: **(A)** pRGEB32, **(B)** pBun421 and **(C)** JD633. NTC: non-transformed control plant, generated from embryos through tissue culture along with transformed embryos.

The transgene integration was verified by plasmid-specific primer pairs designed within the T-DNA region (**Figure 4**; **Supplementary Table. S2**). The transformation efficiency was very low for pRGEB32-based vectors. We could regenerate PCR positive plants only in the background of Reedling. It was also low with the pBun421-based vectors, but we could regenerate plants in Fielder at an efficiency of 0.2 ± 0.1%. Further, we could regenerate some plants from Reedling and Kachu, but none were PCR positive. Inclusion of the GRF4-GIF1 chimera increased our transformation efficiency to 10.6 ± 2.2% for Fielder while it ranged from 4.6% - 12.8% for other cultivars (**Figure 3B**; **Table 4**). The average transformation efficiency for Fielder, Reedling, Baj and Kachu, combined together, increased nearly 60-fold with JD633-based vectors in comparison to pBun423-based vectors. In some batches of Fielder, we achieved up to 41% transformation efficiency. The GRF4-GIF1 chimera thus had significant impact on both regeneration and transformation efficiencies (**Figures 3A, B**). The transgene copy number varied from three to sixteen for Fielder and one to thirteen for Reedling (**Supplementary Table S5**). Regardless, we were able to obtain many transgene-free lines in T1 generation (**Supplementary Figure S1**). For example, 11 copies of the transgene were apparently integrated in each of the lines *lr67-57* and *lr67-60*. Yet, we recovered one transgene-free plant from the eight, we screened in the T1 generation (**Supplementary Figure S1**). Similarly, we obtained two transgene-free T1 plants for the *lr67-60* line, one of which was homozygous for the two mutant alleles, *lr67-4B* and *4D*. In T2 generation of the line *lr67-57*, we recovered two transgene-free plants, which were each homozygous for the two mutant alleles, *lr67-4A* and *lr67-4B.* Similarly, we recovered transgene-free triple homozygous *mlo* mutants for five Fielder and two Reedling lines (**Supplementary Figure S2**).

**Table 4:** Average transformation efficiency of different cultivars after transformation with pBun421- and JD633 (carrying GRF4-GIF1 growth regulation chimera)-based constructs as confirmed by PCR.

Cultivar	PBun421 plasmids^*^			GRF4-GIF1 carrying plasmids^*^		
	Mean (%)	SEM	N^#^	Mean (%)	SEM	N
Fielder	0.20	0.14	22	10.57	2.24	20
Reedling	0.00	0.00	3	8.24	1.49	9
Kachu	0.00	0.00	2	4.57	0.00	1
Baj	0.00	0.00	2	7.00	6.33	2
Morocco	NA			8.47	2.77	3
RL6077	NA			12.80	0.00	1
Sujata	NA			7.20	3.20	2

^*^Mean transformation efficiency values are significantly different between PBun421 plasmids and GRF4-GIF1 carrying plasmids for the same cultivar (P<0.0001).

N: Number of experimental batches with 100 – 200 calli per batch

NA, No experiment was performed.

### Gene editing

**Figure 5:**
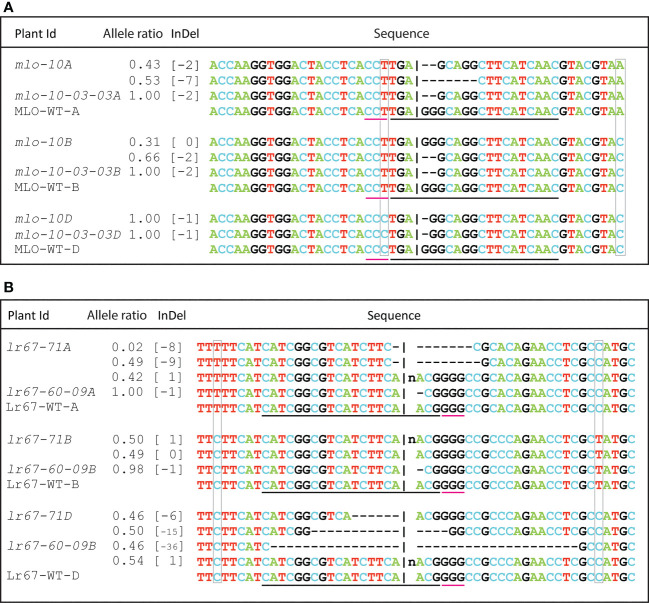
Analysis of mutants by Sanger sequencing: The overlapping peaks of heterozygous mutants were deconvoluted and compared with wild type gene sequence by Inference of CRISPR Edits **(ICE)** analysis. Multiple alleles of single plant (heterozygous) are presented in separate lines under the same plant Id. WT: Wild type. The black underline indicates the location of the guide RNA spacer sequence, and the red underline is for the position of the PAM motif. The natural polymorphisms among the homoeologs have been marked with gray boxes. **(A)** mutation pattern of all three homoeologs of a T0 plant *mlo-10* and its T2 offspring, **(B)** Allelic information of two different *Lr67*-edited lines.

The transgenic plants generated using the pRGEB32-based vector did not show any mutation even in the T4 generation. In contrast, we observed triple mutant lines for *TamloA, TamloB and TamloD* using both pBun421 and JD633-based vectors (**Supplementary Figure S3A**). All 61 T0 plants analyzed so far showed triple edits in Fielder, Reedling and Baj. The T0 plants were either homozygous or heterozygous for mutations (**Figure 5A**, **Supplementary Figure S3A**). Some of the mutant lines were advanced to T2 generation. The target sequences were amplified, and the mutations were further reconfirmed by Sanger sequencing. The targeted editing was inherited and reconfirmed in T2 plants (**Figure 5A**). The corresponding Sanger chromatograms are presented in **Supplementary Datasheet S1**. The uniqueness of the homoeologs is highlighted with gray boxes around the nucleotides that differentiate the alleles (**Figure 5A**). Most of the observed mutations in the *MLO* alleles were upstream of the PAM site, which is underlined with a red line that extends from the black line that corresponds to the guide RNA sequence (**Figure 5A**).

For the *Lr67* gene, we were able to obtain mutations only for the *lr67-4B* and 4*D* alleles with the pBun421-based constructs, and those too only in Fielder. With the JD633-based constructs, in contrast, we recovered mutations in all three homeoalleles, *lr67-4A, 4B* and *4D*, in Fielder, Kachu, Morocco, Reedling, RL6077 and Sujata. Some of the lines contained edits for two or all three of the homeoalleles in the same plant (**Supplementary Figure S3D-F**; **Figure 5B**; **Supplementary Datasheet S1**).

### Phenotype, transgene inheritance and segregation

**Figure 6:**
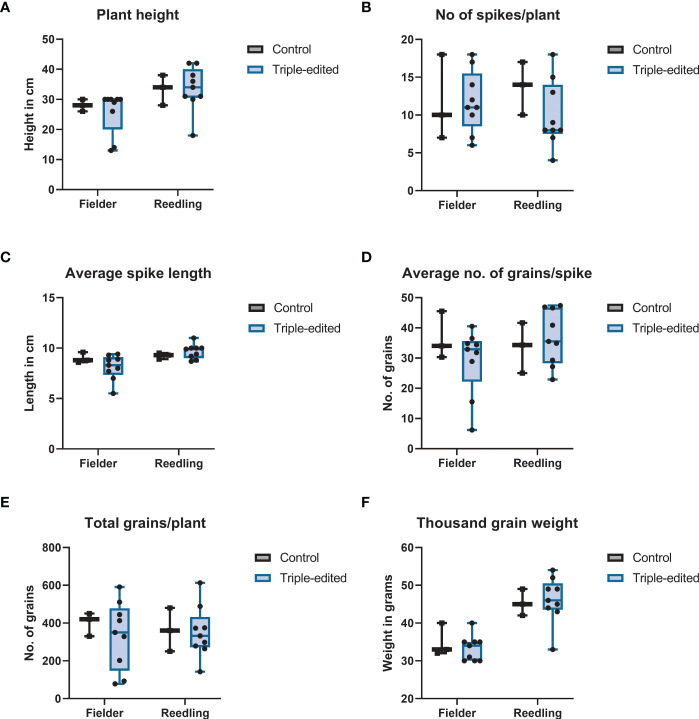
Box plot of mutant phenotypes at the time of maturity: **(A)** plant height, **(B)** No of spikes per plant, **(C)** average spike length, **(D)** average number of grains per spike, **(E)** total number of grains per plant and **(F)** thousand grain weight extrapolated from measurement of 100 seeds. None of the traits showed any statistically significant differences between the gene-edited and non-transformed control plants.

All transgenic plants produced in this study had normal plant height, spike length, number of seeds per spike and thousand grain weight in the greenhouse (**Figure 6**). To verify germline inheritance of the transgene, 20-50 T1 seeds from five different lines were germinated for each construct. Non-transformed control seeds were used as a negative control. The T-DNA inheritance was screened by PCR. Despite high copy number integration, we observed many T1 plants with the edited genes segregated away from the transgene.

## Discussion

We demonstrate, in this study, a wheat transformation protocol that requires fewer than 90 days to regenerate plantlets. We further demonstrate its utility by transforming seven different bread wheat cultivars and editing genes in six of them.

A combination of two different auxins, 2,4-D (2,4-dichlorophenoxyacetic acid) and picloram (4-Amino-3,5,6-trichloro-2-pyridine-carboxylic acid) enhanced callus induction and somatic embryogenesis as compared to 2,4-D alone. Though Jordan (2000) used only 2,4-D (2 mg/L) for regeneration of plantlets, Slamet-Leodin et al. (2014) used a combination of 2,4-D (1 mg/L), NAA (1 mg/L) and 6-benzylaminopurine (BAP) (0.2 mg/L) for callus induction. Similarly, He et al. (2010) and Hayta et al. (2019) used a combination of 2,4-D and picloram to improve somatic embryogenesis in durum and bread wheat. The concentration and time of application play a role. Although He et al. (2010) reported that the use of 10 mg/L and 2 mg/L picloram in co-cultivation and callus induction media, respectively, resulted in higher transformation efficiency, we observed declining regeneration once its concentration exceeded 1 mg/L. Once callus induction has occurred, reduction or removal of auxins from the culture medium is required to allow the establishment of the polar auxin gradient and embryogenesis (Schiavone and Cooke, 1987). This may have been the reason for the declining regeneration efficiencies with higher concentration of picloram in callus induction media in our experiments.

After the first callus induction for 28 days in dark, a second callus induction and selection in the dark for two weeks greatly improved the regeneration efficiency. Similar to our observation, inhibition of somatic embryogenesis of carrot under continuous light has also been documented (Smith and Krikorian, 1989).

As an alternative to the traditional approach of manipulating hormone combinations and concentrations, the expression of plant developmental regulators has been shown to increase the embryogenesis and regeneration efficiency in various crop plants. Some of the examples include *LEAFY COTYLEDON1* (*LEC1*) (Lotan et al., 1998) and *LEAFY COTYLEDON2* (*LEC2*) (Stone et al., 2008) in Arabidopsis, *WUSCHEL* (*WUS*) (Zuo et al., 2002) in maize and *BABY BOOM* (*BBM*) in Arabidopsis and brassica (Boutilier et al., 2002). Overexpression of the maize *BBM* and *WUS2* genes substantially increased the transformation frequencies in maize, sorghum, sugarcane, and indica rice (Lowe et al., 2016; Lowe et al., 2018). The wheat *GRF4–GIF1* chimera significantly improved the regeneration efficiency of our calli in seven different wheat cultivars. We achieved 4.6-12.8% transformation efficiency. This chimera was previously shown to improve the regeneration efficiency using *Agrobacterium*-mediated transformation of Fielder and other wheat varieties (Debernardi et al., 2020).

All the transgenic plants carrying the T-DNA overexpressing the *GRF4–GIF1* chimera were fertile and phenotypically normal in the greenhouse. Debernardi et al. (2020) observed a slight change in the number of grains per spike and grain weight but we did not observe any difference (**Figure 6**). Since the end-product in gene editing is transgene-free, GRF4-GIF1 affecting other traits should not be of concern. The plasmids pBun421 and JD633 carry different selection markers. While both pRGEB32 and JD633 carry the same selection marker, *HygR*, the selection marker gene is driven by different promoters and might have partially affected the selection and regeneration efficiency. The integration site of the transgene is also important for its expression. Regardless, our ultimate objective was to edit genes in elite wheat cultivars, which we have accomplished with this protocol. Even though the transgene copy number varied widely, the segregation in T1 generation and recovery of transgene-free homozygous single and double edits from a relatively small number of segregating plants suggests that concatenated copies of the transgene were likely integrated instead of random insertions across the genome (Svitashev et al., 2002; Smirnov and Battulin, 2021).

The wheat transgenic plants generated using the pRGEB32-based vector did not show any mutation even after advancing them to T4 generation. These results differ from those previously reported where no mutations were observed in early generations but were only identified in subsequent generations in Bobwhite (Wang et al., 2018). In contrast to the current study in wheat, using the same vector system in rice, triple mutants were generated for three *OsXLG* genes with high editing efficiency (Biswal et al., 2022).

We observed homozygous triple mutants for the *TaMLO* gene in T0 generation using pBun421- and JD633-based vectors. This observation suggests that for gene editing in wheat, the TaU3 or TaU6 promoter driving the expression of the gRNA cassette is superior to the OsU3 promoter. Although TaU3 and OsU3 promoters were earlier reported to be similar for gene editing in maize protoplasts (Xing et al., 2014), in agreement with our experience, Howells et al. (2018) also failed to edit genes in wheat by using the OsU3 promoter. We observed single, double, and triple mutants (non-homozygous) of the *Lr67* homeoalleles using the JD633-based vector, whereas only single and double mutants were obtained with the pBun421-based vectors. We are currently in the process of phenotyping the single and stacked mutant alleles of *lr67* for rust resistance and the triple homozygous *mlo* mutants for powdery mildew resistance.

## Conclusions

We standardized a protocol for the transformation of commercial wheat cultivars, which were previously recalcitrant to transformation. Incorporation of the wheat GRF4-GIF1 growth regulator chimera significantly improved the regeneration efficiency. The transformation frequency increased nearly 60-fold for the selected cultivars. With this protocol, we edited genes directly in elite wheat cultivars. TaU3 or TaU6, as opposed to OsU3, promoter to drive the gRNA expression was key in obtaining nearly perfect gene editing efficiency.

## Data availability statement

The original contributions presented in the study are included in the article/**Supplementary Material**. Further inquiries can be directed to the corresponding author.

## Author contributions

AB and KD conceptualized the work, AB developed the gene constructs, designed tissue culture experiments, carried out molecular characterization, analyzed, and interpreted the data and wrote the manuscript. LB and AR performed transformation experiments and helped with molecular characterization. JD helped in designing the constructs, and critically revising the manuscript. KD identified the target genes, provided overall guidance to the project, and critically revised the manuscript. All authors contributed to the article and approved the submitted version.

## Supplementary material

Supplementary Figure 1 Segregation of the transgene in T1 generation: **(A)** PCR amplification using the transgene specific primers (pRGEB32_7045F/pRGEB32_8155R) in Fielder (lr67-07 and lr67-41) and Reedling (lr67-57) lines, **(B)** PCR amplification of above plants using wheat gene *Lr67*-specific primers (Lr67A_923F/Lr67A_1876R) as PCR controls. The CIM024 plasmid was used as positive control for transgene verification while Fielder and Reedling genomic DNA served as negative controls. For the PCR control reaction in **(B)**, Fielder and Reedling genomic DNA was used as positive control while the plasmid served as negative control.

Supplementary Figure 2 Segregation of the transgene in T2 generation for the MLO CRISPR lines: **(A)** PCR amplification using the transgene specific primers (pRGEB32_7045F/pRGEB32_8155R) in Fielder (mlo-01-05-04, mlo-03-02-04, mlo-04-05-01, mlo-09-02-02, mlo-09-02-04, mlo-10-03-03, mlo-10-05-01and mlo-10-05-06) and Reedling (mlo-17-05-01 and mlo-18-05-02) lines, **(B)** PCR amplification of above plants using wheat gene *mlo*-specific primers (MLO_487F/MLOA_1722R) as PCR controls. The CIM026 plasmid was used as positive control for transgene verification while Fielder and Reedling genomic DNA served as negative controls. For the PCR control reaction in **(B)**, Fielder and Reedling genomic DNA was used as positive control while the plasmid served as negative control.

Supplementary Figure 3 Surveyor assay for mutation detection in target genes: **(A)** mutation analysis of *MLO-A*, *B* and *D* homoeologs (similar results were obtained for both pBun421 and JD633-based vectors; **(B, C)** mutation analysis for *Lr67-B* and *D* genomes for plants developed using pBun421 vector (no mutant was observed for *Lr67-A* homoelog); **(D–F)** mutation analysis for *Lr67-A*, *B* and *D* homoeologs for plants developed using JD633-based vectors. Note: Mutants have been labelled in italics with small fonts.

Supplementary Table 1 List of guides used and oligos synthesized for developing constructs.

Supplementary Table 2 Primers used for verification of plasmid integration and transgene copy number.

Supplementary Table 3 Primers for amplifying DNA sequences flanking the target sequence that was analyzed by Surveyor nuclease assay.

Supplementary Table 4 Raw data on transformation, regeneration, transformation, and gene editing.

Supplementary Table 5 Determination of transgene copy number by real-time quantitative PCR (qPCR).

## References

[B1] Bhalla P. L. (2006). Genetic engineering of wheat - current challenges and opportunities. Trends Biotechnol. 24, 305–311. doi: 10.1016/j.tibtech.2006.04.008 16682090

[B2] Biswal A. K. Wu T.-Y. Urano D. Pelissier R. Morel J.-B. Jones A. M.. (2022). Novel mutant alleles reveal a role of the extra-Large G protein in rice grain filling, panicle architecture, plant growth, and disease resistance. Front. Plant Sci. 12. doi: 10.3389/fpls.2021.782960 PMC8761985 35046975

[B3] Boutilier K. Offringa R. Sharma V. K. Kieft H. Ouellet T. Zhang L.. (2002). Ectopic expression of BABY BOOM triggers a conversion from vegetative to embryonic growth. Plant Cell 14, 1737–1749. doi: 10.1105/tpc.001941 12172019 PMC151462

[B4] Cheng M. Fry J. E. Pang S. Zhou H. Hironaka C. M. Duncan D. R.. (1997). Genetic transformation of wheat mediated by agrobacterium tumefaciens. Plant Physiol. 115, 971–980. doi: 10.1104/pp.115.3.971 12223854 PMC158560

[B5] Conant D. Hsiau T. Rossi N. Oki J. Maures T. Waite K.. (2022). Inference of CRISPR edits from Sanger trace data. Cris. J. 5, 123–130. doi: 10.1089/crispr.2021.0113 35119294

[B6] Cram D. Kulkarni M. Buchwaldt M. Rajagopalan N. Bhowmik P. Rozwadowski K.. (2019). WheatCRISPR: a web-based guide RNA design tool for CRISPR/Cas9-mediated genome editing in wheat. BMC Plant Biol. 19, 474. doi: 10.1186/s12870-019-2097-z 31694550 PMC6836449

[B7] Debernardi J. M. Tricoli D. M. Ercoli M. F. Hayta S. Ronald P. Palatnik J. F.. (2020). A GRF–GIF chimeric protein improves the regeneration efficiency of transgenic plants. Nat. Biotechnol. 38, 1274–1279. doi: 10.1038/s41587-020-0703-0 33046875 PMC7642171

[B8] Dehairs J. Talebi A. Cherifi Y. Swinnen J. V. (2016). CRISP-ID: decoding CRISPR mediated indels by Sanger sequencing. Sci. Rep. 6, 28973. doi: 10.1038/srep28973 27363488 PMC4929496

[B9] Dhugga K. S. (2022). Gene editing to accelerate crop breeding. Front. Plant Sci. 13. doi: 10.3389/fpls.2022.889995 PMC9196881 35712601

[B10] Ding L. Li S. Gao J. Wang Y. Yang G. He G. (2009). Optimization of agrobacterium-mediated transformation conditions in mature embryos of elite wheat. Mol. Biol. Rep. 36, 29–36. doi: 10.1007/s11033-007-9148-5 17906943

[B11] Garbus I. Romero J. R. Valarik M. Vanžurová H. Karafiátová M. Cáccamo M.. (2015). Characterization of repetitive DNA landscape in wheat homeologous group 4 chromosomes. BMC Genomics 16, 375. doi: 10.1186/s12864-015-1579-0 25962417 PMC4440537

[B12] Hawkesford M. J. (2014). Reducing the reliance on nitrogen fertilizer for wheat production. J. Cereal Sci. 59, 276–283. doi: 10.1016/j.jcs.2013.12.001 24882935 PMC4026125

[B13] Hayta S. Smedley M. A. Demir S. U. Blundell R. Hinchliffe A. Atkinson N.. (2019). An efficient and reproducible agrobacterium-mediated transformation method for hexaploid wheat (Triticum aestivum l.). Plant Methods 15, 1–15. doi: 10.1186/s13007-019-0503-z 31673278 PMC6815027

[B14] He Y. Jones H. D. Chen S. Chen X. M. Wang D. S. W. Li K. X.. (2010). Agrobacterium-mediated transformation of durum wheat (Triticum turgidum l. var. durum cv Stewart) with improved efficiency. J. Exp. Bot. 61, 1567–1581. doi: 10.1093/jxb/erq035 20202997 PMC2852660

[B15] Herrera-Foessel S. A. Lagudah E. S. Huerta-Espino J. Hayden M. J. Bariana H. S. Singh D.. (2011). New slow-rusting leaf rust and stripe rust resistance genes Lr67 and Yr46 in wheat are pleiotropic or closely linked. Theor. Appl. Genet. 122, 239–249. doi: 10.1007/s00122-010-1439-x 20848270

[B16] Herrera-Foessel S. A. Singh R. P. Lillemo M. Huerta-Espino J. Bhavani S. Singh S.. (2014). Lr67/Yr46 confers adult plant resistance to stem rust and powdery mildew in wheat. Theor. Appl. Genet. 127, 781–789. doi: 10.1007/s00122-013-2256-9 24408377

[B17] Hiei Y. Komari T. (2006). Improved protocols for transformation of indica rice mediated by agrobacterium tumefaciens. Plant Cell. Tissue Organ Cult. 85, 271–283. doi: 10.1007/s11240-005-9069-8

[B18] Hiei Y. Komari T. (2008). Agrobacterium-mediated transformation of rice using immature embryos or calli induced from mature seed. Nat. Protoc. 3, 824–834. doi: 10.1038/nprot.2008.46 18451790

[B19] Howells R. M. Craze M. Bowden S. Wallington E. J. (2018). Efficient generation of stable, heritable gene edits in wheat using CRISPR/Cas9. BMC Plant Biol. 18, 215. doi: 10.1186/s12870-018-1433-z 30285624 PMC6171145

[B20] Ishida Y. Hiei Y. Komari T. (2015a). “High efficiency wheat transformation mediated by agrobacterium tumefaciens,” in Advances in wheat genetics: from genome to field. Eds. Ogihara Y. Takumi S. Handa H. (Tokyo: Springer Japan), 167–173. doi: 10.1007/978-4-431-55675-6_18

[B21] Ishida Y. Tsunashima M. Hiei Y. Komari T. (2015b). “Wheat (Triticum aestivum l.) transformation using immature embryos,” in Agrobacterium protocols, vol. 1 . Ed. Wang K. (New York, NY:Springer New York), 189–198. doi: 10.1007/978-1-4939-1695-5_15 25300841

[B22] Ismagul A. Yang N. Maltseva E. Iskakova G. Mazonka I. Skiba Y.. (2018). A biolistic method for high-throughput production of transgenic wheat plants with single gene insertions. BMC Plant Biol. 18, 1–8. doi: 10.1186/s12870-018-1326-1 29940859 PMC6020210

[B23] Jordan M. C. (2000). Green fluorescent protein as a visual marker for wheat transformation. Plant Cell Rep. 19, 1069–1075. doi: 10.1007/s002990000246 30754772

[B24] Kan J. Cai Y. Cheng C. Jiang C. Jin Y. Yang P. (2022). Simultaneous editing of host factor gene TaPDIL5-1 homoeoalleles confers wheat yellow mosaic virus resistance in hexaploid wheat. New Phytol. 234, 340–344. doi: 10.1111/nph.18002 35092005

[B25] Li S. Lin D. Zhang Y. Deng M. Chen Y. Lv B.. (2022). Genome-edited powdery mildew resistance in wheat without growth penalties. Nature 602, 455–460. doi: 10.1038/s41586-022-04395-9 35140403

[B26] Liu Y. Luo W. Linghu Q. Abe F. Hisano H. Sato K.. (2021). In planta genome editing in commercial wheat varieties. Front. Plant Sci. 12. doi: 10.3389/fpls.2021.648841 PMC8006942 33790930

[B27] Lotan T. Ohto M. A. Matsudaira Yee K. West M. A. L. Lo R. Kwong R. W.. (1998). Arabidopsis LEAFY COTYLEDON1 is sufficient to induce embryo development in vegetative cells. Cell 93, 1195–1205. doi: 10.1016/S0092-8674(00)81463-4 9657152

[B28] Lowe K. La Rota M. Hoerster G. Hastings C. Wang N. Chamberlin M.. (2018). Rapid genotype “independent” zea mays l. (maize) transformation via direct somatic embryogenesis. Vitr. Cell. Dev. Biol. - Plant 54, 240–252. doi: 10.1007/s11627-018-9905-2 PMC5954046 29780216

[B29] Lowe K. Wu E. Wang N. Hoerster G. Hastings C. Cho M. J.. (2016). Morphogenic regulators baby boom and wuschel improve monocot transformation. Plant Cell 28, 1998–2015. doi: 10.1105/tpc.16.00124 27600536 PMC5059793

[B30] Miroshnichenko D. Ashin D. Pushin A. Dolgov S. (2018). Genetic transformation of einkorn (Triticum monococcum l. ssp. monococcum l.), a diploid cultivated wheat species 06 biological sciences 0604 genetics. BMC Biotechnol. 18, 1–13. doi: 10.1186/s12896-018-0477-3 30352590 PMC6199808

[B31] Moore J. W. Herrera-Foessel S. Lan C. Schnippenkoetter W. Ayliffe M. Huerta-Espino J.. (2015). A recently evolved hexose transporter variant confers resistance to multiple pathogens in wheat. Nat. Genet. 47, 1494–1498. doi: 10.1038/ng.3439 26551671

[B32] Oerke E. C. (2006). Crop losses to pests. J. Agric. Sci. 144, 31–43. doi: 10.1017/S0021859605005708

[B33] Piffanelli P. Zhou F. Casais C. Orme J. Jarosch B. Schaffrath U.. (2002). The barley MLO modulator of defense and cell death is responsive to biotic and abiotic stress stimuli. Plant Physiol. 129, 1076–1085. doi: 10.1104/pp.010954 12114562 PMC166502

[B34] Pixley K. V. Falck-Zepeda J. B. Paarlberg R. L. Phillips P. W. B. Slamet-Loedin I. H. Dhugga K. S.. (2022). Genome-edited crops for improved food security of smallholder farmers. Nat. Genet. 54, 364–367. doi: 10.1038/s41588-022-01046-7 35393597

[B35] Qiu F. Xing S. Xue C. Liu J. Chen K. Chai T.. (2022). Transient expression of a TaGRF4-TaGIF1 complex stimulates wheat regeneration and improves genome editing. Sci. China Life Sci. 65, 731–738. doi: 10.1007/s11427-021-1949-9 34406572

[B36] Raghurami-Reddy M. Acaso J. T. Alakonya A. E. Mangrauthia S. K. Sundaram R. M. Balachandran S. M.. (2022). “Accelerating cereal breeding for disease resistance through genome editing,” in Genome editing technologies for crop improvement (Singapore: Springer Nature Singapore), 323–347. doi: 10.1007/978-981-19-0600-8_15

[B37] Rajendrakumar P. Biswal A. K. Balachandran S. M. Ramesha M. S. Viraktamath B. C. Sundaram R. M. (2007). A mitochondrial repeat specific marker for distinguishing wild abortive type cytoplasmic male sterile rice lines from their cognate isogenic maintainer lines. Crop Sci. 47, 207–211. doi: 10.2135/cropsci2006.06.0365

[B38] Richardson T. Thistleton J. Higgins T. J. Howitt C. Ayliffe M. (2014). Efficient agrobacterium transformation of elite wheat germplasm without selection. Plant Cell. Tissue Organ Cult. 119, 647–659. doi: 10.1007/s11240-014-0564-7

[B39] Rustgi S. Ankrah N. O. Brew-Appiah R. A. T. Sun Y. Liu W. von Wettstein D. (2017). “Doubled Haploid Transgenic Wheat Lines by Microspore Transformation,” in Wheat Biotechnology, Methods Mol. Biol. Eds. P. L. Bhalla and M. B. Singh (New York, NY: Humana Press), 213–234. doi: 10.1007/978-1-4939-7337-8_13 28913803

[B40] Schiavone F. M. Cooke T. J. (1987). Unusual patterns of somatic embryogenesis in the domesticated carrot: developmental effects of exogenous auxins and auxin transport inhibitors. Cell Differ. 21, 53–62. doi: 10.1016/0045-6039(87)90448-9 3607884

[B41] Shepherd C. T. Lauter A. N. M. Scott,. M. P. (2009). “Determination of Transgene Copy Number by Real-Time Quantitative PCR,” in Transgenic Maize, Methods Mol. Biol., Ed. Scott M. P. (Totowa, NJ: Humana Press) 129–134. doi: 10.1007/978-1-59745-494-0_11 19378009

[B42] Singh P. Kumar K. (2022). Agrobacterium-mediated in-planta transformation of bread wheat (Triticum aestivum l.). J. Plant Biochem. Biotechnol. 31, 206–212. doi: 10.1007/s13562-021-00669-x

[B43] Slamet-Loedin I. H. Chadha-Mohanty P. Torrizo L. (2014). “Agrobacterium-mediated transformation: rice transformation,” in Cereal genomics: methods and protocols, methods in molecular biology. Eds. Henry R. J. Furtado A. (Totowa, NJ: Humana Press), 261–271. doi: 10.1007/978-1-62703-715-0_21 24243210

[B44] Smirnov A. Battulin N. (2021). Concatenation of transgenic DNA: random or orchestrated? Genes (Basel). 12, 1969. doi: 10.3390/genes12121969 34946918 PMC8701086

[B45] Smith D. L. Krikorian A. D. (1989). Release of somatic embryogenic potential from excised zygotic embryos of carrot and maintenance of proembryonic cultures in hormone-free medium. Am. J. Bot. 76, 1832–1843. doi: 10.1002/j.1537-2197.1989.tb15172.x 11540921

[B46] Stone S. L. Braybrook S. A. Paula S. L. Kwong L. W. Meuser J. Pelletier J.. (2008). Arabidopsis LEAFY COTYLEDON2 induces maturation traits and auxin activity: implications for somatic embryogenesis. Proc. Natl. Acad. Sci. U. S. A. 105, 3151–3156. doi: 10.1073/PNAS.0712364105/SUPPL_FILE/12364FIG9.JPG 18287041 PMC2268600

[B47] Supartana P. Shimizu T. Nogawa M. Shioiri H. Nakajima T. Haramoto N.. (2006). Development of simple and efficient in planta transformation method for wheat (Triticum aestivum l.) using agrobacterium tumefaciens. J. Biosci. Bioeng. 102, 162–170. doi: 10.1263/jbb.102.162 17046528

[B48] Svitashev S. K. Pawlowski W. P. Makarevitch I. Plank D. W. Somers D. A. (2002). Complex transgene locus structures implicate multiple mechanisms for plant transgene rearrangement. Plant J. 32, 433–445. doi: 10.1046/j.1365-313X.2002.01433.x 12445116

[B49] Tanaka J. Minkenberg B. Poddar S. Staskawicz B. Cho M.-J. (2022). Improvement of gene delivery and mutation efficiency in the CRISPR-Cas9 wheat (Triticum aestivum l.) genomics system *via* biolistics. Genes (Basel). 13, 1180. doi: 10.3390/genes13071180 35885963 PMC9318839

[B50] Tassy C. Barret P. (2017). “Biolistic transformation of wheat,” in Methods Mol. Biol., Eds. Bhalla P. L. Singh M. B. (New York, NY: Humana Press) 141–152. doi: 10.1007/978-1-4939-7337-8_9 28913799

[B51] Vasil V. Castillo A. M. Fromm M. E. Vasil I. K. (1992). Herbicide resistant fertile transgenic wheat plants obtained by microprojectile bombardment of regenerable embryogenic callus. Nat. Biotechnol. 10, 667–674. doi: 10.1038/nbt0692-667

[B52] Wang Y. Cheng X. Shan Q. Zhang Y. Liu J. Gao C.. (2014). Simultaneous editing of three homoeoalleles in hexaploid bread wheat confers heritable resistance to powdery mildew. Nat. Biotechnol. 32, 947–951. doi: 10.1038/nbt.2969 25038773

[B53] Wang W. Pan Q. He F. Akhunova A. Chao S. Trick H.. (2018). Transgenerational CRISPR-Cas9 activity facilitates multiplex gene editing in allopolyploid wheat. Cris. J. 1, 65–74. doi: 10.1089/crispr.2017.0010 PMC6319321 30627700

[B54] Wu H. Sparks C. Amoah B. Jones H. D. (2003). Factors influencing successful agrobacterium-mediated genetic transformation of wheat. Plant Cell Rep. 21, 659–668. doi: 10.1007/s00299-002-0564-7 12789416

[B55] Wulff B. B. H. Dhugga K. S. (2018). Wheat–the cereal abandoned by GM. Science 361, 451–452. doi: 10.1126/science.aat5119 30072526

[B56] Xie K. Minkenberg B. Yang Y. (2015). Boosting CRISPR/Cas9 multiplex editing capability with the endogenous tRNA-processing system. Proc. Natl. Acad. Sci. 112, 3570–3575. doi: 10.1073/pnas.1420294112 25733849 PMC4371917

[B57] Xing H.-L. Dong L. Wang Z.-P. Zhang H.-Y. Han C.-Y. Liu B.. (2014). A CRISPR/Cas9 toolkit for multiplex genome editing in plants. BMC Plant Biol. 14, 327. doi: 10.1186/s12870-014-0327-y 25432517 PMC4262988

[B58] Zale J. M. Agarwal S. Loar S. Steber C. M. (2009). Evidence for stable transformation of wheat by floral dip in agrobacterium tumefaciens. Plant Cell Rep. 28, 903–913. doi: 10.1007/s00299-009-0696-0 19308413 PMC2688021

[B59] Zuo J. Niu Q.-W. Frugis G. Chua N.-H. (2002). The WUSCHEL gene promotes vegetative-to-embryonic transition in arabidopsis. Plant J. 30, 349–359. doi: 10.1046/j.1365-313X.2002.01289.x 12000682

