# Supplementary material for: An efficient transformation method for genome editing of elite bread wheat cultivars
Source: Front Plant Sci. 2023 May 16;14:1135047. doi: 10.3389/fpls.2023.1135047 (PMC10234211; doi:10.3389/fpls.2023.1135047)
Supplement: Supplementary file 1 [file DataSheet_1.pdf]

SUPPLEMENTARY DATASHEET 1: ANALYSIS OF MUTANTS BY SANGER SEQUENCING AND INFERENCE OF CRISPR EDITS (ICE)

A) Analysis of *mlo* mutants. The sequence at the top shows the mutation predicted by ICE analysis (Conant et al., 2022). The upper chromatogram is for the mutant plant while the lower chromatogram is for the control parent line.

mlo-10A: Hetero

```
0.4300 [-2] ACCAAGGTGGACTACCTCACCTTGA|--GCAGGCTTCATCAACGTACGTAATACCCCAAA
0.5300 [-7] ACCAAGGTGGACTACCTCACCTTGA|-------CTTCATCAACGTACGTAATACCCCAAA
```

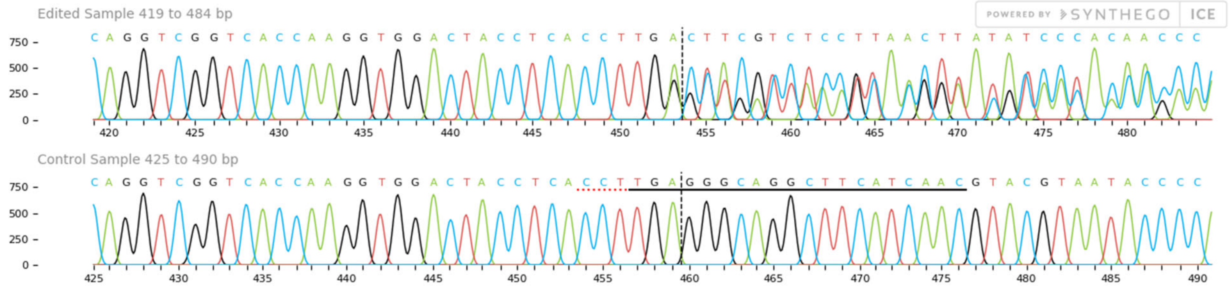

mlo-10B: Hetero

```
0.3100 [ 0] ACCAAGGTGGACTACCTCACCTTGA|GGGCAGGCTTCATCAACGTACGTACAACCAAAAAAA
0.6600 [-2] ACCAAGGTGGACTACCTCACCTTGA|--GCAGGCTTCATCAACGTACGTACAACCAAAAAAA
```

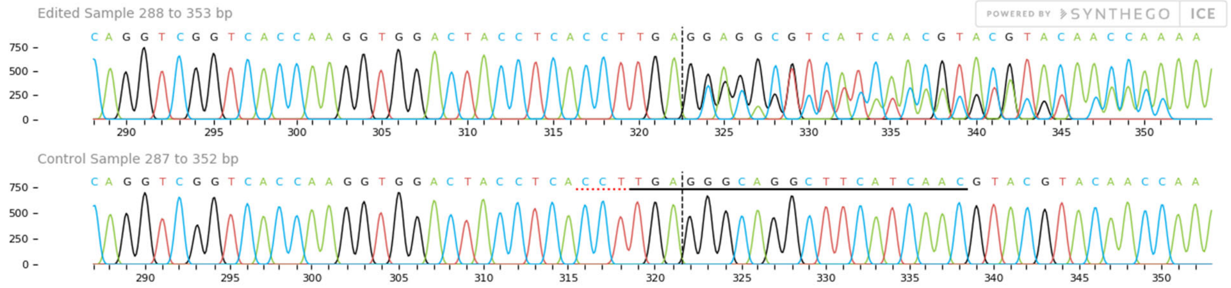

mlo-10D: Homo

```
1.0000 [-1] ACCAAGGTGGACTACCTCACCCTGA|-GGCAGGCTTCATCAACGTACGTACCAAAACAAA
```

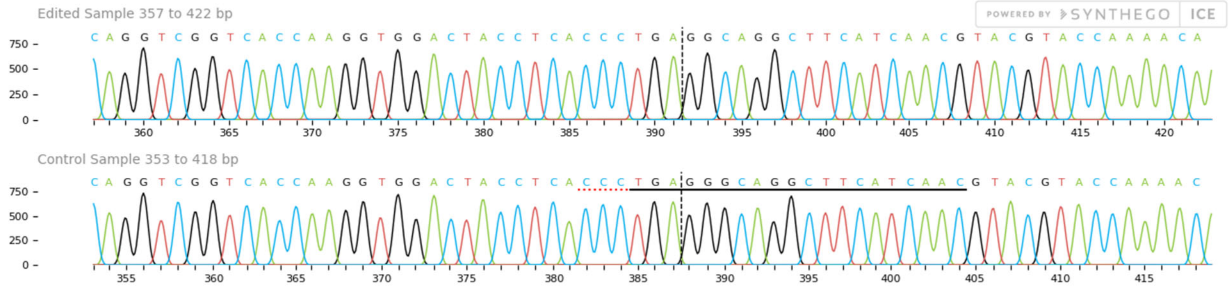

mlo-10-03-03A: Homo

```
1.0000 [-2] ACCAAGGTGGACTACCTCACCTTGA|--GCAGGCTTCATCAACGTACGTAATACCCCAAAAG
```

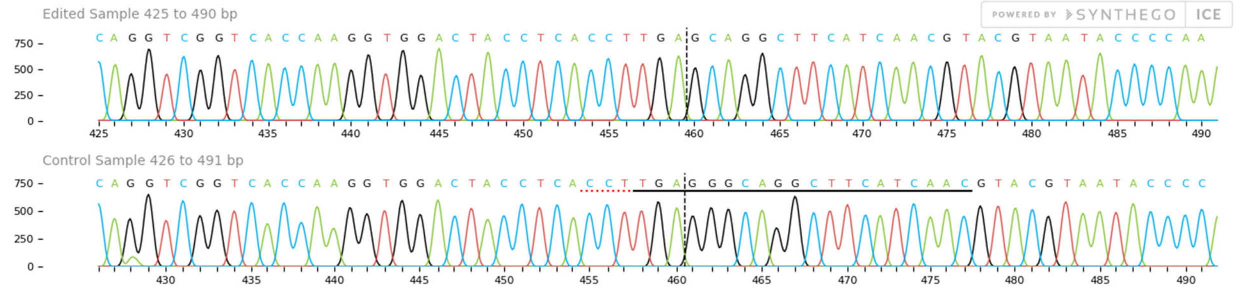

mlo-10-03-03B: Homo

```
1.0000 [-2] ACCAAGGTGGACTACCTCACCTTGA|--GCAGGCTTCATCAACGTACGTACAACCAAAAAAAG
```

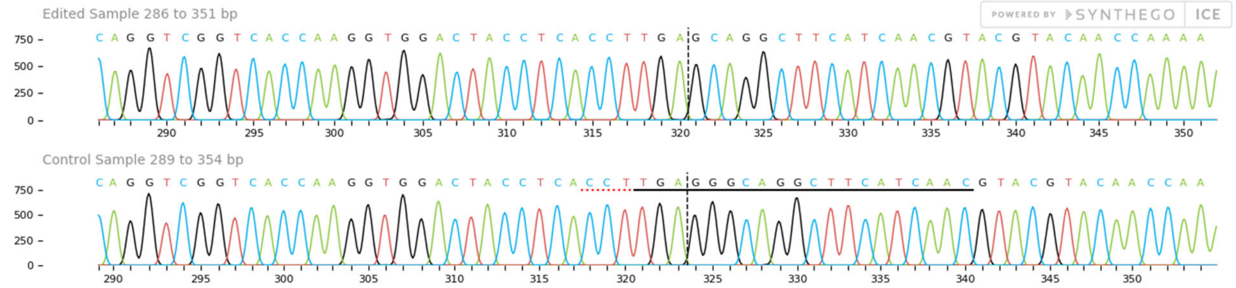

mlo-10-03-03D: Homo

```
1.0000 [-1] ACCAAGGTGGACTACCTCACCCTGA|-GGCAGGCTTCATCAACGTACGTACCAAAACAAATCC
```

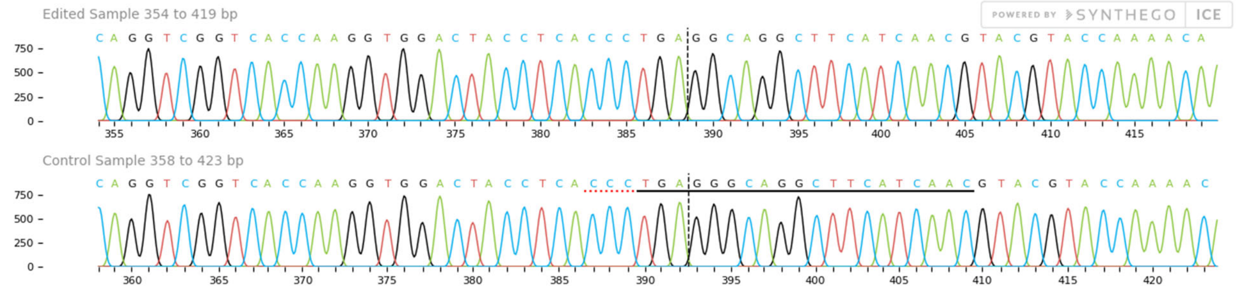

B) Analysis of two triple heterozygous *Lr67* mutants. The sequence on the top shows the kind of mutation that was predicted ICE analysis (Conant et al., 2022). The upper chromatogram refers to the test plant while the lower chromatogram represents the non-transformed control parent line.

Lr67-71 A: Hetero

```
0.0200 [-8] TTTTTCATCATCGGCGTCATCTTC-|-------CGCACAGAACCTCGCCATGCTCATCATCGGC
0.4900 [-9] TTTTTCATCATCGGCGTCATCTTC-|--------GCACAGAACCTCGCCATGCTCATCATCGGC
0.4200 [1]  TTTTTCATCATCGGCGTCATCTTCA|nACGGGGCCGCACAGAACCTCGCCATGCTCATCATCGG
```

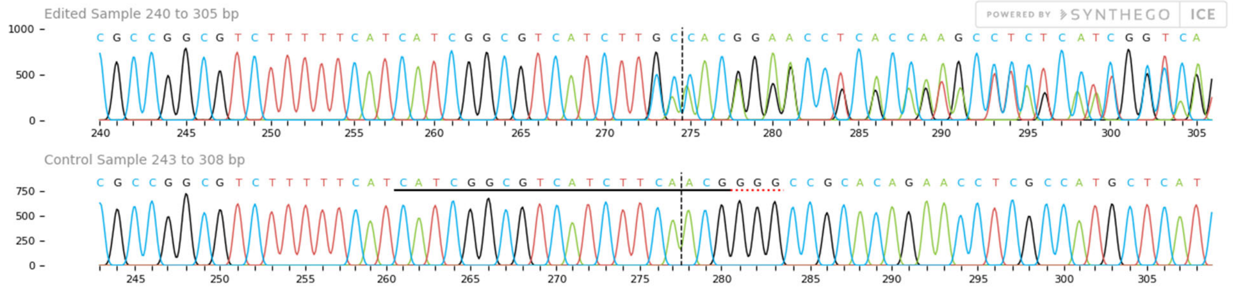

Lr67-71 B: Hetero

```
0.5000 [1]    TTCTTCATCATCGGCGTCATCTTCA|nACGGGGCCGCCCAGAACCTCGCTATGCTTATCATCG
0.4900 [1]    TTCTTCATCATCGGCGTCATCTTCA| ACGGGGCCGCCCAGAACCTCGCTATGCTTATCATCG
```

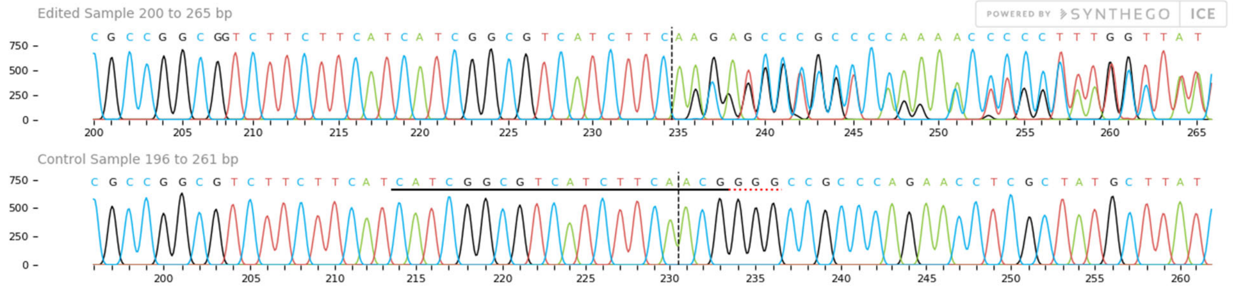

Lr67-71 D: Hetero

```
0.4600 [ -6] TTCTTCATCATCGGCGTCA------|ACGGGGCCGCCCAGAACCTCGCCATGCTCATCATCG
0.5000 [-15] TTCTTCATCATCGG-----------|----GGCCGCCCAGAACCTCGCCATGCTCATCATCG
```

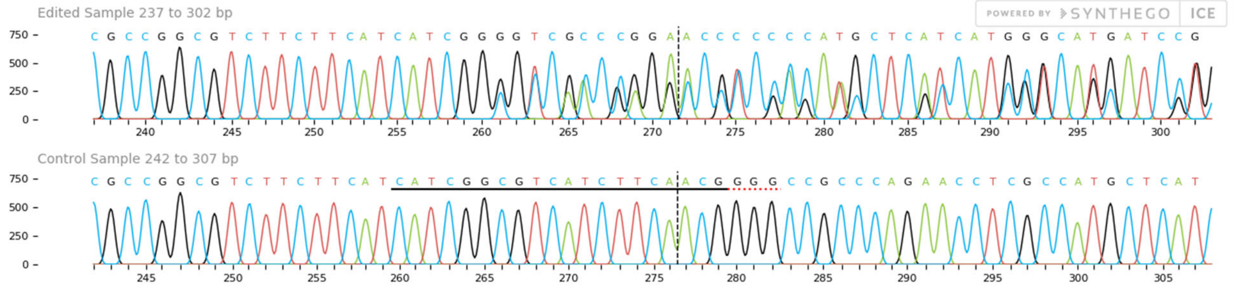

Lr67-60-09A Homo

```
1.0000 [-1] TTTTTCATCATCGGCGTCATCTTCA|-CGGGGCCGCACAGAACCTCGCCATGCTCATCAT
```

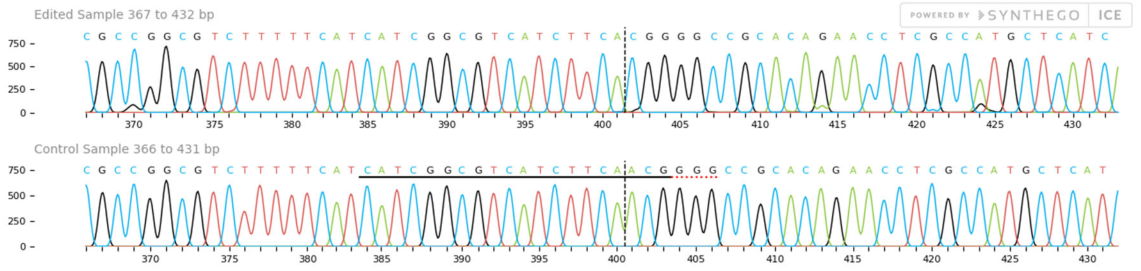

Lr67-60-09B Homo

```
0.9800 [-1] TTCTTCATCATCGGCGTCATCTTCA|-CGGGGCCGCCCAGAACCTCGCTATGCTTATCAT
```

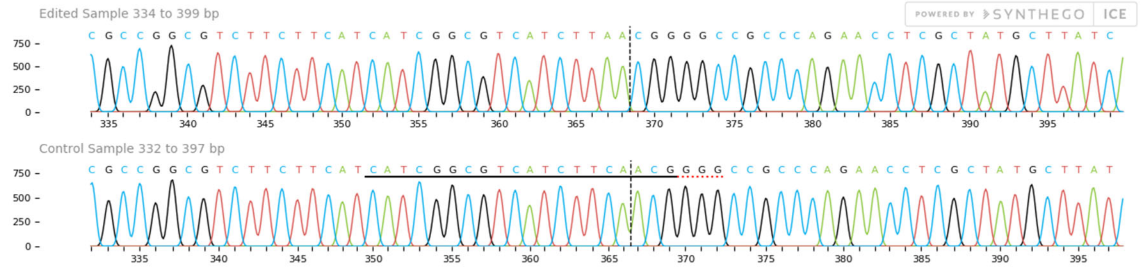

Lr67-60-09D Hetero

```
0.4600 [-36] TTCTTCATC----------------| --------------------GCCATGCTCAT
0.5400 [1]   TTCTTCATCATCGGCGTCATCTTCA|nACGGGGCCGCCCAGAACCTCGCCATGCTCAT
```

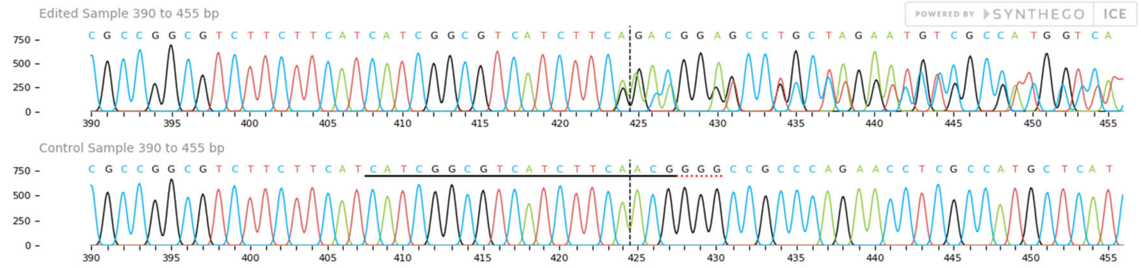

REFERENCE:

Conant, D., Hsiau, T., Rossi, N., Oki, J., Maures, T., Waite, K., et al. (2022). Inference of CRISPR Edits from Sanger Trace Data. *Cris. J.* 5, 123–130. doi:10.1089/crispr.2021.0113.
