# Supplementary material for: An efficient transformation method for genome editing of elite bread wheat cultivars
Source: Front Plant Sci. 2023 May 16;14:1135047. doi: 10.3389/fpls.2023.1135047 (PMC10234211; doi:10.3389/fpls.2023.1135047)
Supplement: Supplementary Figure 2 — Segregation of the transgene in T2 generation for the MLO CRISPR lines: (A) PCR amplification using the transgene specific primers (pRGEB32_7045F/pRGEB32_8155R) in Fielder (mlo-01-05-04, mlo-03-02-04, mlo-04-05-01, mlo-09-02-02, mlo-09-02-04, mlo-10-03-03, mlo-10-05-01and mlo-10-05-06) and Reedling (mlo-17-05-01 and mlo-18-05-02) lines, (B) PCR amplification of above plants using wheat gene mlo-specific primers (MLO_487F/MLOA_1722R) as PCR controls. The CIM026 plasmid was used as positive control for transgene verification while Fielder and Reedling genomic DNA served as negative controls. For the PCR control reaction in (B), Fielder and Reedling genomic DNA was used as positive control while the plasmid served as negative control. [file Image_2.pdf]

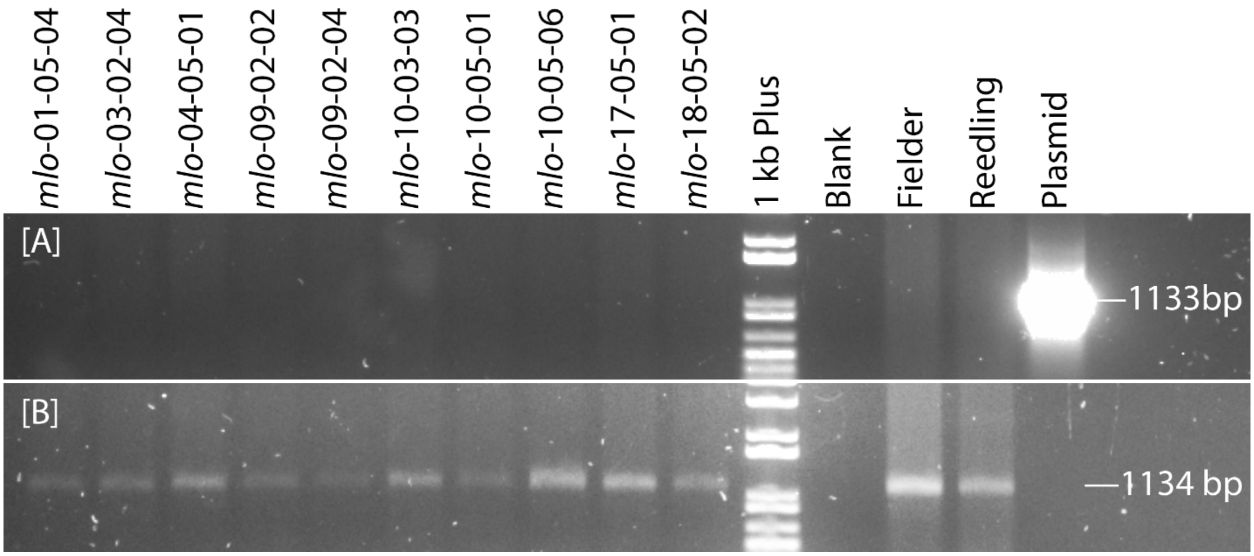

Supplementary Figure S2: Segregation of the transgene in T2 generation for the MLO CRISPR lines: A) PCR amplification using the transgene specific primers (pRGEB32_7045F/ pRGEB32_8155R) in Fielder (*mlo-01-05-04*, *mlo-03-02-04*, *mlo-04-05-01*, *mlo-09-02-02*, *mlo-09-02-04*, *mlo-10-03-03*, *mlo-10-05-01* and *mlo-10-05-06*) and Reedling (*mlo-17-05-01* and *mlo-18-05-02*) lines, B) PCR amplification of above plants using wheat gene *mlo*-specific primers (MLO_487F/ MLOA_1722R) as PCR controls. The CIM026 plasmid was used as positive control for transgene verification while Fielder and Reedling genomic DNA served as negative controls. For the PCR control reaction in (B), Fielder and Reedling genomic DNA was used as positive control while the plasmid served as negative control.
