# Supplementary material for: An efficient transformation method for genome editing of elite bread wheat cultivars
Source: Front Plant Sci. 2023 May 16;14:1135047. doi: 10.3389/fpls.2023.1135047 (PMC10234211; doi:10.3389/fpls.2023.1135047)
Supplement: Supplementary Figure 3 — Surveyor assay for mutation detection in target genes: (A) mutation analysis of MLO-A, B and D homoeologs (similar results were obtained for both pBun421 and JD633-based vectors; (B, C) mutation analysis for Lr67-B and D genomes for plants developed using pBun421 vector (no mutant was observed for Lr67-A homoelog); (D–F) mutation analysis for Lr67-A, B and D homoeologs for plants developed using JD633-based vectors. Note: Mutants have been labelled in italics with small fonts. [file Image_3.pdf]

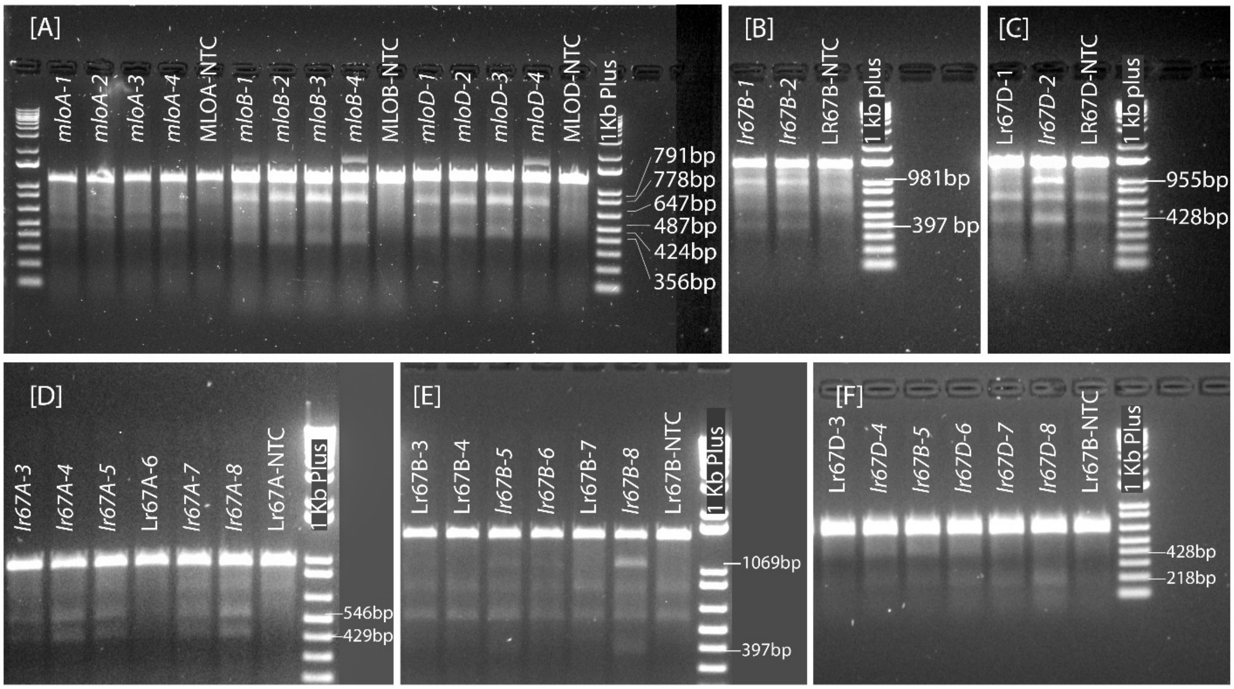

Supplementary Figure S3: Surveyor assay for mutation detection in target genes: A) mutation analysis of *MLO-A*, *B* and *D* homoeologs (similar results were obtained for both pBun421 and JD633-based vectors; B and C) mutation analysis for *Lr67-B* and *D* genomes for plants developed using pBun421 vector (no mutant was observed for *Lr67-A* homoelog); D-F) mutation analysis for *Lr67-A*, *B* and *D* homoeologs for plants developed using JD633-based vectors.

Note: Mutants have been labelled in italics with small fonts
