# Supplementary material for: An efficient transformation method for genome editing of elite bread wheat cultivars
Source: Front Plant Sci. 2023 May 16;14:1135047. doi: 10.3389/fpls.2023.1135047 (PMC10234211; doi:10.3389/fpls.2023.1135047)
Supplement: Supplementary Table 1 — List of guides used and oligos synthesized for developing constructs. [file Table_1.pdf]

Supplementary Table S1: List of guides used and oligos synthesized for developing constructs

Guide name	Sequence	Vector used	Oligo name	Sequence	Construct name	Target gene
LR67cr2	gccggtggccgccatgatgc	pRGEB32	LR67cr2	Commercially cloned at Genscript	CIM014	Lr67 (all homoeologs)
Lr67cr9	catcggcgtcatcttcaacg	pBun421	LR67cr9BsaIF	agcgatcggcgtcatcttcaacg	CIM019	Lr67 (all homoeologs)
			LR67cr9BsaIR	aaaccgttgaagatgacgccgat		
		JD633	LR67cr9AarIF	acttcatcggcgtcatcttcaacg	CIM024	Lr67 (all homoeologs)
			LR67cr9AarIR	aaaccgttgaagatgacgccgatg		
MLOcr22	gttgatgaagcctgccctca	pBun421	MLOcr22BsaIF	agcgttgatgaagcctgccctca	CIM020	MLO (all homoeologs)
			MLOcr22BsaIR	aaactgagggcaggcttcatcaa		
		JD633	MLOcr22AarIF	acttgttgatgaagcctgccctca	CIM026	MLO (all homoeologs)
			MLOcr22AarIR	aaactgagggcaggcttcatcaac		
