# Supplementary material for: An efficient transformation method for genome editing of elite bread wheat cultivars
Source: Front Plant Sci. 2023 May 16;14:1135047. doi: 10.3389/fpls.2023.1135047 (PMC10234211; doi:10.3389/fpls.2023.1135047)
Supplement: Supplementary Table 2 — Primers used for verification of plasmid integration and transgene copy number. [file Table_2.pdf]

Supplementary Table S2: Primers used for verification of plasmid integration and transgene copy number

**Primer pair**	**Sequence (5'-3')**	**Ta (°C)**	**Amplicon size (bp)**	**Target Region**	**Target plants**
PRGEB_10939F	tgccatgggttggtgcaaacac	64.0	1142	Ubi Promoter – Cas9 CDS	CIM014
PRGEB_12080R	tggtaggccacctcgtccacg				
35S_755F	aagggatgacgcacaatccca	60.0	1012	35S promoter – Hyg CDS	CIM014
HptIIR	agctgcatcatcgaaattgccgtc				
35S_755F	aagggatgacgcacaatccca	58.0	764	35S Promoter -Blp CDS	CIM019, CIM020
pRGEB32_7045F	tgctcaacacatgagcgaaacc				
TaU6_246F	aatggaaagctgatgcacgga	58.0	695	TaU3/U6 Promoter -gRNA region	CIM019, CIM020
pBun2242R	tcggctgtcttgtcacagagaa				
TaU6_851F	gcttttaggcccgcatgatcg	59.0	304	TaU3/U6 Promoter -gRNA region	CIM24, CIM026
pRGEB32_389R	cagggttttcccagtcacgacg				
pRGEB32_7045F	tgctcaacacatgagcgaaacc	59.0	1133	Hyg CDS – CaMVPoly A signal	CIM24, CIM026
pRGEB32_8155R	tgaactcaccgcgacgtctgtc				
TaU6_851F	gcttttaggcccgcatgatcg	60	243	TaU6 Promoter - gRNA region	CIM24, CIM026
pRGEB_gRNAR	cgactcggtgccactttttca				
Lr67_1369F	ctttcacagcgtccagctcga	60	95	First intron-Second Exon region	CIM24, CIM026
Lr67OEP_2R	acgcaggaaatcgtccatcga				
