# Supplementary material for: An efficient transformation method for genome editing of elite bread wheat cultivars
Source: Front Plant Sci. 2023 May 16;14:1135047. doi: 10.3389/fpls.2023.1135047 (PMC10234211; doi:10.3389/fpls.2023.1135047)
Supplement: Supplementary Table 3 — Primers for amplifying DNA sequences flanking the target sequence that was analyzed by Surveyor nuclease assay. [file Table_3.pdf]

Supplementary Table S3: Primers for amplifying DNA sequences flanking the target sequence that was analyzed by Surveyor nuclease assay

Construct	Forward	Sequence (5-3')	Target (flanking region)	Amplicon size (bp)	Left frag (bp)	Right Frag (bp)
CIM014	Lr67A_486F	ctctggagaaacactcgctgc	Lr67A	450	163	283
	Lr67A_950R	ctcctctcttccatgctggctc				
	Lr67B_321F	ctgcggagaaacactcgctgc	Lr67B	421	156	265
	Lr67B_950R	ctcctctctttcatgcgtggtt				
	Lr67D_486F	ctgtggagaaacactcgctgc	Lr67D	383	156	227
	Lr67D_950R	ctcctctctttcatacgcgcgg				
CIM019	Lr67A_923F	tgtacacctccagatcacgtc	Lr67A	986	429	558
	Lr67A_1908R	cgacgtacctacatactgcagc				
	Lr67D_1241F	agctgtcttttgtgtggacacg	Lr67B	1377	397	981
	Lr67B_2235R	cggaaaattatgtctcgcgca				
	Lr67D_1241F	agctgtcttttgtgtggacacg	Lr67D	1382	428	955
	Lr67D_2622R	cagccacaccagcaaactctgc				
CIM024	Lr67A_923F	tgtacacctccagatcacgtc	Lr67A	975	429	546
	Lr67A_1876R	catactgcagctagcgtgagta				
	Lr67D_1241F	agctgtcttttgtgtggacacg	Lr67B	1466	397	1069
	Lr67B_2324R	ttctttgatgctaccgtggac				
	Lr67D_1241F	agctgtcttttgtgtggacacg	Lr67D	646	428	218
	Lr67D_1886R	acttcggtttctaccaacacc				
CIM020<br>CIM026	MLO_487F	tcatcatggctctaagccgtc	MLOA	1134	487	647
	MLOA_1722R	tcaaatggggtttggctgttgc				
	MLOB_590F	ttaacctgccccggcgtactc	MLOB	1146	356	791
	MLOB_1736R	cttgttgctgggctcaaccacg				
	MLOD_703F	gtcaggcctacctgttcatgc	MLOD	1202	424	778
	MLOB_1736R	cttgttgctgggctcaaccacg				
