# Supplementary material for: An efficient transformation method for genome editing of elite bread wheat cultivars
Source: Front Plant Sci. 2023 May 16;14:1135047. doi: 10.3389/fpls.2023.1135047 (PMC10234211; doi:10.3389/fpls.2023.1135047)
Supplement: Supplementary Table 4 — Raw data on transformation, regeneration, transformation, and gene editing. [file Table_4.pdf]

Supplementary Table S4: Raw data on transformation, regeneration, transformation, and gene editing

Vector	Number of batches	Number of embryos transformed	Number of regenerated plants	Number of PCR positive plants	Number of edited plants/Number of plants tested	Comments
pRGEB32	49	14,081	318	8	0/8	None of the plants showed editing.
pBun421	29	5,490	47	8	8/8	Two plants were double edited for Lr67, and six plants were triple edited for MLO in T_0 generation. All plants were Fielder and the editing efficiency for at least one gene in each plant = 100%.
JD633	42	5,605	563	492	145/160	For MLO, the editing efficiency was 100% for 31 Fielder, 10 Reedling and 21 Baj analyzed plants. All plants were triple edited.<br><br>For Lr67, 4 of 5 APAV#1 (80%), 50 of 54 Fielder (93%), 5 of 6 Kachu (83%), 13 of 17 Morocco (76%), 6 of 10 Reedling (60%), 2 of 2 RL6077 (100%) and 3 of 4 Sujata (75%) T_0 plants were mutant for at least one homeolog.
