# Supplementary material for: An efficient transformation method for genome editing of elite bread wheat cultivars
Source: Front Plant Sci. 2023 May 16;14:1135047. doi: 10.3389/fpls.2023.1135047 (PMC10234211; doi:10.3389/fpls.2023.1135047)
Supplement: Supplementary Table 5 — Determination of transgene copy number by real-time quantitative PCR (qPCR). [file Table_5.pdf]

Supplementary Table S5: Calculation of transgene copy number by qPCR

Plant ID	Parent Genotype	C_T Mean (transgene)	C_T SD (Transgene)	C_T Mean (Reference)	C_T SD (Reference)	Copy Number
lr67-07	Fielder	24.69	0.11	24.79	0.12	3
lr67-17	Fielder	21.71	0.07	24.12	0.34	16
lr67-28	Fielder	23.71	0.14	24.82	0.36	6
lr67-34	Fielder	24.22	0.34	24.48	0.39	4
lr67-41	Fielder	22.91	0.20	24.31	0.30	8
lr67-42	Fielder	24.54	0.14	24.62	0.27	3
lr67-54	Reedling	24.79	0.01	24.98	0.27	3
lr67-55	Reedling	22.47	0.08	24.56	0.09	13
lr67-57	Reedling	22.59	0.23	24.52	0.13	11
lr67-60	Reedling	22.19	0.30	24.06	0.05	11
lr67-63	Reedling	24.28	0.61	23.88	0.12	2
lr67-65	Reedling	25.10	0.56	23.69	0.27	1
lr67-71	Reedling	22.83	0.44	24.06	0.29	7
Control	Fielder	38.07	1.13	23.78	0.16	0
Control	Reedling	37.22	0.99	23.39	0.00	0
